# Induced Pluripotent Stem Cells as a Promising Path Toward Zone-Specific Meniscus Regeneration

**DOI:** 10.3390/cells15211961

**Published:** 2026-10-28

**Authors:** Florence Fioretti, Guoqiang Hua, Nadia Benkirane-Jessel, Rana Smaida

**Affiliations:** 1 INSERM UMR1260, Nanomédecine Régénérative, 1 Rue Eugène Boeckel, 67000 Strasbourg, France; f.fioretti@unistra.fr (F.F.); g.hua@unistra.fr (G.H.); 2 Université de Strasbourg, Faculté de Chirurgie Dentaire, Faculté de Médecine, 4 R Blaise Pascal, 67000 Strasbourg, France; 3 Hôpitaux Universitaire de Strasbourg, 1 place de l’Hôpital, 67000 Strasbourg, France

**Keywords:** induced pluripotent stem cells, meniscus regeneration, zonal heterogeneity, fibrochondrocyte, biofabrication, vascularization, tissue engineering, regenerative medicine

## Abstract

**Highlights:**

**What are the main findings?**

- Current treatments for irreparable meniscal tears, partial meniscectomy and allograft transplantation leave a gap: no approach yet restores native, zone-specific meniscal tissue, and iPSCs offer a scalable potential solution if directed toward zone-specific cells.
- Building a functional living meniscus requires zone-specific matrix composition and maturation, anisotropic collagen architecture, and root-anchored anatomical integration, and direct iPSC-meniscus evidence to support this remains limited to a single short-term rabbit study.

**What is the implication of the main finding?**

- Closing this gap requires combinatorial marker panels validated against native zonal references, biofabrication strategies that reproduce zone-specific matrix composition and mechanics, and indication-specific requirements for focal, partial, and whole-meniscus replacement.
- Clinical translation and regulatory qualification will depend on measurable, staged go/no-go criteria covering safety, potency, and manufacturing, since the approach remains a research platform rather than an established therapy.

**Abstract:**

For young and middle-aged patients with irreparable meniscal tears, preserving or restoring meniscal tissue is critical to maintaining joint function and delaying osteoarthritis. Yet, when repair is not feasible, partial meniscectomy remains common and can compromise the load-bearing function of the knee, underscoring a highly unmet medical need for regenerative strategies that restore functional tissue rather than simply relieve symptoms. Induced pluripotent stem cells (iPSCs) offer a scalable and potentially patient-matched source of progenitor cells for meniscal regeneration, with iPSC-derived cartilage already entering clinical study for articular cartilage defects. However, translating this progress to the meniscus presents a fundamental biological challenge: restoring a specialized, zonally organized tissue rather than simply generating sufficient cells. The meniscus is a structurally, mechanically, and biologically graded tissue with distinct inner, transitional, and outer zones. Consequently, generating fibrocartilage is unlikely to reproduce native meniscal function. In this review, we examine iPSC-based strategies through the framework of zone-specific meniscal regeneration, focusing on meniscal fibrochondrocyte generation and reconstruction of native tissue architecture. We compare biofabrication strategies against native structural, vascular, and mechanical benchmarks, and outline the preclinical, manufacturing and regulatory requirements for translating iPSC-derived meniscal constructs toward patient-centered clinical application.

## 1. Introduction

Meniscal injury is common; meniscus surgery is one of the most commonly performed orthopedic procedures worldwide, which can compromise long-term knee function [1,2]. Its consequences extend beyond the acute injury, because loss of meniscal tissue can compromise load distribution and contribute to progressive joint degeneration [3,4]. Tears occur across the lifespan, from young athletes to older adults [1,3], and much of the damage is clinically silent. In a Framingham (Massachusetts) community sample, meniscal tear or destruction prevalence on knee MRI rose with age, from 19% in women aged 50–59 to 56% in men aged 70–90; 61% of those with a tear reported no pain, aching or stiffness in the prior month, and among knees with radiographic osteoarthritis, tear prevalence was 63% with frequent pain versus 60% without [5], so many tears are incidental rather than symptom-causing. The meniscus is essential to knee mechanics: its wedge-shaped, hydrated body converts axial load into circumferential hoop stress via collagen bundles anchored at the anterior and posterior roots, increasing tibiofemoral contact area and protecting articular cartilage from excessive stress [2,3,6]. This matters most for young and middle-aged patients with irreparable inner-zone tears, where partial meniscectomy, the common treatment when repair is not feasible, relieves symptoms at the cost of load-bearing tissue [2].

Because the inner zone is poorly vascularized, tears there have limited healing potential and are frequently treated by resection [3]. Meniscectomy and disruption of the meniscal roots alter contact mechanics, increase articular cartilage loading, and are associated with subsequent joint degeneration [4,6,7]. For these patients, the therapeutic gap is particularly important: the joint is damaged, but the patient is often decades away from being a candidate for arthroplasty, while no established procedure regenerates the lost meniscal tissue. The FIDELITY and METEOR trials both found arthroscopic partial meniscectomy no better than sham surgery or physical therapy for degenerative tears [8,9]. Meniscal allograft transplantation (MAT) remains the only established option for total meniscal replacement, with favorable long-term survival reported in selected young patients [2,10,11], but its broader clinical use is constrained by limited graft availability, the need for accurate size matching, and considerations around graft fixation and surgical technique [2,10,12]. This population, patients with symptomatic meniscal tissue loss whose knees are not yet suitable for arthroplasty, therefore represents a clinically important target for regenerative medicine. The tissue itself presents a fundamental biological challenge because the meniscus is graded rather than uniform. It is conventionally divided by vascularity into an outer (red-red) zone, an intermediate (red-white) zone and a relatively avascular inner (white-white) zone [3]. These vascular zones correlate with the compositional and cellular gradient across the tissue: the outer region contains fibroblast-like cells in a type I collagen matrix, whereas the inner region contains rounded, chondrocyte-like fibrochondrocytes in a matrix richer in type II collagen and aggrecan [2,3], although transcriptomically they remain distinct from articular chondrocytes [13].

Throughout this review, outer, transitional and inner refer to the compositional gradient, and red-red, red-white and white-white to vascularity. Vascularity is itself graded rather than binary: micro-CT of six cadaveric menisci (mean donor age 75 years) found vessels in every transverse layer, and ISAKOS has discouraged the red/white nomenclature for describing tears [14]. Regional differences in cellularity, vascularity and proteoglycan content are already present in the developing meniscus and change during childhood [15]. Accordingly, successful regeneration requires more than restoring tissue volume or generating a generic cartilaginous phenotype; it requires reconstruction of the meniscus as a zonally organized tissue, including its cellular identity, extracellular matrix composition, collagen architecture and mechanical function. Any regenerative strategy would need to reproduce this zonal organization, together with the collagen alignment and the root attachments through which hoop stress develops.

Despite substantial progress in tissue engineering, reliable restoration of this complex tissue remains difficult. Acellular and decellularized scaffold approaches have not resolved the gap between templating a shape and regenerating a functional tissue [16,17,18]. Cell-based tissue engineering has advanced considerably but still relies on adult cell sources, which share the limits of scarcity, donor-to-donor variability and phenotype loss on expansion [2,13,19,20,21]. These limitations highlight the need for a cell source that is both scalable and capable of being precisely instructed toward the specialized phenotypes required for meniscal regeneration. 

iPSCs address the cell-supply constraint that has limited regenerative strategies. Reprogramming of somatic cells to pluripotency with defined factors provides a renewable, patient-matchable source of cells that can be driven toward cartilaginous phenotypes [22,23,24], and iPSC-derived hyaline cartilage has progressed from proof of concept to large-animal studies and, for articular cartilage, a first-in-human study registered in Japan (jRCTa050190104) [25,26,27,28,29]. This trajectory provides an important, though early, proof of principle for the therapeutic potential of iPSC-derived cartilage, but translating this progress to the meniscus presents a fundamentally different challenge. Pluripotent stem cell-based approaches have reached meniscus patients through intra-articular administration of hESC-derived immunity-and-matrix-regulatory cells (IMRCs) in a phase I trial [30]. This cell-therapy strategy differs from structural tissue replacement. Direct in vivo evidence for an iPSC-derived cartilage graft in meniscal repair remains limited to a short-term rabbit study, which demonstrated cell engraftment but variable repair-tissue phenotypes [31]. The key translational challenge is therefore to develop iPSC-derived constructs that reproduce zone-specific meniscal identity, architecture and mechanical function.

This review addresses how iPSCs can be deployed, which biofabrication technologies can impose meniscal architecture, why zone-specific fibrochondrocytes are a more appropriate differentiation target than hyaline chondrocytes, how a construct of clinically relevant size could be supplied with nutrients, and what preclinical, clinical and regulatory steps remain.

Several recent reviews cover cartilage tissue engineering, meniscus tissue engineering and cell sources for iPSC-based cartilage products [27,32,33,34]. Meniscal zonation and vascularity [2,3,35], stem cell sources [2,3], biofabrication [33,34] and translational barriers [2] have each been addressed in earlier reviews. To our knowledge, this is the first review to integrate iPSC differentiation biology with zone-specific meniscal identity and functional reconstruction. Its main added value is this integration, not the individual topics, and its scope and conclusions differ from the recent reviews above. Nordberg et al. [27] cover articular cartilage engineering across diarthrodial joints (knee, hip, temporomandibular and facet joints), with emphasis on cell-based products, inflammatory environments and the translational pathway; they conclude that new cartilage repair products are being enabled by advances in cell sources, stimuli and scaffolds, and meniscus appears only in passing. Bandyopadhyay et al. [32] review 3D printing, injectable hydrogels and physical stimulation for meniscal repair, including design, fixation and lubrication, and conclude that these three approaches are the main emerging routes to meniscal tissue engineering and that combining them could yield treatments that clear regulatory hurdles; the cell sources they consider are adult cells (fibrochondrocytes, chondrocytes, and bone marrow, synovial, adipose and meniscus-derived mesenchymal cells), and iPSCs are not discussed. Li et al. [33] review implantable hydrogels for meniscal repair and conclude with the challenges of hydrogel use. Eremeev et al. [34] address iPSC-based products for articular cartilage, focusing on donor-cell source, epigenetic memory and disease-associated differences in chondrogenic potential, and conclude that these factors need systematic study. Our own previous review, Ali et al. [36], covers iPSC chondrogenic differentiation in general, including disease modeling, drug screening and manufacturing; it treats fibrocartilage as an undesired outcome of differentiation and refers to zone-specific function only for articular cartilage grafts, and it concludes that protocol optimization, genome editing, 3D bioprinting and automated manufacturing are needed. The present review reaches a different central conclusion: generating cartilage is not sufficient, and the decisive problem is the controlled specification of inner, transitional and outer meniscal fibrochondrocytes and their assembly into a root-anchored, mechanically graded construct.

This review additionally connects zone-specific differentiation to the developmental biology of the synovial joint interzone and to the tenogenic as well as chondrogenic arms of iPSC differentiation and incorporates the meniscal root problem, vascular constraints in centimeter-scale constructs, and an explicit framework of potential failure modes.

This is a narrative rather than a systematic review. PubMed, Scopus and Web of Science were searched from inception to 16 October 2026, supplemented by hand-searching of the reference lists of retrieved articles and trial registries (ClinicalTrials.gov, Japan Registry of Clinical Trials (jRCT)). The search strings combined: (‘induced pluripotent stem cell’ OR ‘iPSC’ OR ‘pluripotent’) AND (‘meniscus’ OR ‘meniscal’ OR ‘fibrochondrocyte’); (‘meniscus’ AND (‘zonal’ OR ‘inner zone’ OR ‘outer zone’ OR ‘circumferential’ OR ‘tensile modulus’)); (‘iPSC’ OR ‘pluripotent’) AND (‘chondrogenesis’ OR ‘chondrocyte’ OR ‘cartilage’) AND (‘hypertrophy’ OR ‘SOX9’ OR ‘GDF5’ OR ‘joint interzone’); (‘meniscus’ OR ‘cartilage’) AND (‘biofabrication’ OR ‘bioprinting’ OR ‘melt electrowriting’ OR ‘scaffold’); and (‘pluripotent’ OR ‘cell therapy’) AND (‘tumorigenicity’ OR ‘potency assay’ OR ‘advanced therapy medicinal product’ OR ‘regulatory’). English-language primary studies and reviews on these topics were included; conference abstracts and preprints were not. Search results were screened for relevance to the scope of this narrative review. The authors screened search results against the inclusion and exclusion criteria above, with particular attention to studies addressing iPSC-derived meniscal cells or tissues, meniscal zonal identity, construct architecture, mechanical function, safety and clinical translation. The selection process was intended to identify key primary studies and relevant reviews rather than to provide an exhaustive systematic-review estimate of all available evidence. Where a single primary study provides the only direct evidence for a claim (for example, references [30,31]), this is stated explicitly in the text. 

## 2. From Adult Cells to Pluripotency: Why iPSCs?

The challenge of regenerating a clinically relevant meniscus begins with the cell source. The limits of adult cell sources define the case for pluripotent ones. Autologous meniscal fibrochondrocytes carry the correct, zone-appropriate phenotype but are scarce, require a biopsy of healthy tissue [3], and dedifferentiate on monolayer expansion, with upregulated contractile markers such as ACTA2 (α-smooth-muscle actin) and TAGLN [13] and reduced expression of meniscus-associated genes such as CHAD, CILP and COMP [37]. Articular and costal chondrocytes support self-assembly of mechanically robust neocartilage but still require donor tissue and lose phenotype on expansion [2,21,27].

Mesenchymal stem cells (MSCs) and related cells from bone marrow [38,39], adipose tissue [40] and synovium [41,42] are accessible and chondrogenic. They promoted new matrix formation in avascular meniscal explants [38] and improved meniscal healing in rabbit, rat and primate models [39,40,41,42], yet they are donor-variable [30] and prone to hypertrophy and endochondral drift, in which chondrogenic cells progress to a type X collagen-expressing and mineralizing phenotype [2].

iPSC-derived multipotent cells and adult MSCs also differ in intrinsic chondrogenic potential, so the two routes are not interchangeable [43]. These limitations are: reduced availability, donor dependence, phenotype instability and restricted scalability. It makes it difficult to obtain the cell numbers required for a whole meniscus construct while retaining a defined meniscal identity (Table 1). 

iPSCs offer a fundamentally different starting point: a renewable population that can be expanded before differentiation and subsequently instructed toward specific lineages. Two differentiation strategies are commonly used in skeletal work. In the first, iPSCs pass through a mesenchymal-progenitor state and are then driven to chondrocytes in high-density culture with BMP-2 or TGF-β3; this route uses iPSC-derived mesenchymal progenitors (iMSCs) as the intermediate [44]. In the second, scaffold-free protocols pass through mesendodermal and mixed paraxial/lateral-plate mesoderm populations before nodule formation, and suspension culture yields hyaline cartilaginous particles [25]. Culture substrate (Matrigel^®^ versus laminin 511-E8^®^) influencing cell shape and YAP activity and inactivation around the start of differentiation is associated with efficient chondrogenesis, and transient YAP knockdown restored cartilage formation on laminin. The authors suggest, but did not test, that substrate stiffness is involved in these results [45]. Developmentally guided protocols can steer human pluripotent cells toward articular rather than growth-plate chondrocytes, which matters because growth-plate-like cells are fated to be replaced by bone [46]. These approaches demonstrate that pluripotency can be coupled to increasingly precise control of skeletal cell fate, but they also expose a central limitation for meniscal regeneration: generating a chondrogenic cell is not equivalent to generating a meniscal fibrochondrocyte.

Accordingly, phenotype control is the more difficult problem, and cell number alone does not solve it. Single-cell analysis of human iPSC chondrogenesis showed that standard protocols also generate off-target neural-like and melanocytic cells regulated by WNT signaling and MITF activity and that WNT signaling from these off-target cells promotes chondrocyte hypertrophy. Suppressing these pathways improved yield and homogeneity [47]. Hypertrophy can be limited: when pro-hypertrophic BMPs are withheld and TGF-β is used alone, iPSC-derived chondrocytes then adopt a hypertrophy-resistant, articular-chondrocyte-like phenotype with barely detectable type X collagen [48]. In an hiPSC-sclerotome protocol, BMP inhibition followed by BMP2/GDF5 activation produced chondro-spheroids with minimal COL10A1 and ALP, and cells derived from them formed non-hypertrophic cartilage when transplanted on hyaluronic acid-coated fibrin microbeads, persisting for at least five months in immunocompromised rodents [49]. BMP dependence is protocol-specific. However, the Kyoto protocol requires BMP2, TGF-β1 and GDF5 together [25], and BMPs are usually needed to enhance TGF-β-driven chondrogenesis of iPSC-derived multipotent cells [43]. Thus, the same self-renewal and developmental plasticity that make iPSCs attractive for scalable regeneration also create a requirement for stringent control of lineage purity, maturation and phenotype stability. For meniscal applications, this control must ultimately extend beyond avoiding off-target or hypertrophic fates to actively establishing the zone-specific fibrochondrocyte phenotypes required for functional tissue regeneration. The self-renewal and plasticity that make iPSCs attractive therefore also carry off-target and hypertrophic risks [25,47,48], as well as a theoretical risk of tumor formation, which was not observed in the immunodeficient rodent transplants of iPSC-derived cartilage particles [25].

It is important to mention that the superiority of iPSCs over adult cell sources for meniscal regeneration remains unproven. No study has directly compared iPSC-derived cells with adult meniscal fibrochondrocytes or MSCs for zone-specific meniscal outcomes in the same experimental system; the only direct iPSC-meniscus evidence comes from a separate rabbit study [31], and the only clinical pluripotent-derived meniscal experience to date used hESC-derived cells acting through a paracrine mechanism [30], not patient-matched iPSCs generating tissue. The case for iPSCs is therefore based on their scalability and precise instructability in principle (Figure 1), not on demonstrated functional superiority; establishing that advantage is itself one of the open questions this review identifies.

**Table 1:** Candidate cell sources for meniscus regeneration and their principal trade-offs. MSC, mesenchymal stem cell; ESC, embryonic stem cell; iPSC, induced pluripotent stem cell; iMSC, iPSC-derived MSC; IMRC, immunity-and-matrix-regulatory cell.

Cell Source	Principal Advantage	Principal Limitation	Representative Evidence
Meniscal fibrochondrocytes	Native, zone-appropriate phenotype	Scarce; biopsy of healthy tissue [3]; dedifferentiation on expansion [13]	Aligned scaffolds raised tensile modulus [19]
Articular/costal chondrocytes	Capacity for self-assembly into mechanically robust neocartilage	Donor-limited; phenotype loss on expansion [2,27]	Functional neocartilage formation and anisotropic tissue development [20,21]
Marrow/adipose MSCs	Accessible; chondrogenic	Hypertrophy; donor variability [2,30]	Matrix formation in avascular meniscal explants [38]; improved repair in rabbit and rat defect models [39,40]
Synovial MSCs	Accessible; highly proliferative and chondrogenic	Donor variability; evidence limited to small preclinical studies [41,42]	Better meniscal healing or regeneration in rabbit, microminipig and aged primate models [41,42,50,51]
ESCs	Pluripotent; capable of fibrochondrogenic differentiation [3]	Ethical and immunogenic considerations	hESC-derived IMRCs safe in phase I trial (n = 18); acted mainly on the joint environment [30]
iPSCs/iMSCs	Renewable and potentially patient-matched; no embryo use	Possible tumorigenicity; off-target and hypertrophic differentiation; prolonged differentiation protocols [25,47,48]	iPSC cartilage particles engrafted in rabbit meniscus [31]; iMSC pellets, non-meniscal [27]

## 3. State of the Art: iPSC-Derived Cartilage and Current Meniscal Evidence

Scaffold-free human iPSC-derived cartilage particles survived when transplanted into articular cartilage defects in immunodeficient rats and immunosuppressed minipigs and showed potential for integration with native cartilage, although human cells in the rat defects were replaced by host cells within 8 to 12 weeks [25]. In a primate model, MHC-mismatched allogeneic iPSC-derived cartilage organoids survived, were integrated and remodeled as articular cartilage in knee chondral defects without immunosuppression, with follow-up extending to 17 weeks [26]. iPSC-derived cartilage has also been investigated for another avascular tissue, the nucleus pulposus [52]. iPSCs derived from healthy and osteoarthritic cartilage differ in chondrogenic differentiation, epigenetic regulation and metabolic transcriptomic signatures [53], and animal transplantation studies up to 2021 have been reviewed elsewhere [54]. However, these studies target hyaline articular cartilage and therefore provide only indirect evidence for regeneration of the graded fibrocartilage of the meniscus.

Evidence for pluripotent-derived cells in the meniscus itself is limited to two studies that pursued different strategies: an hESC-derived immunity-and-matrix-regulatory cell (IMRC) study [30] and, in 2026, an iPSC study by Okimura et al. [31]. In the IMRC rabbit experiments, no human cells were detected in the menisci at 8 weeks, consistent with action through host–cell modulation rather than tissue replacement. In contrast, Okimura tested whether pre-formed iPSC-derived hyaline cartilage particles could engraft in meniscus. These two main studies are compared below.

The two studies differ in their experimental designs and intended therapeutic mechanisms and should not be considered directly equivalent. The IMRC study [30] evaluated intra-articular cell injection in a rabbit meniscal-defect model and subsequently in a single-center, open-label, dose-escalation phase I clinical trial. In the rabbit experiment, animals were randomized to intra-articular IMRCs (1 × 10^7^ cells) or saline, all under tacrolimus immunosuppression, and repair was evaluated eight weeks after injection by macroscopic scoring (nine animals per group), histology, collagen staining and Pauli histological scoring (seven animals per group). In the clinical trial, 18 patients with Stoller grade I–II meniscal lesions received one of three doses (1 × 10^7^, 5 × 10^7^ or 1 × 10^8^ cells; six patients per group). The trial had no placebo or untreated clinical control group. Safety was the primary endpoint, while secondary outcomes included VAS pain, WOMAC, Lysholm and American Knee Society scores, together with MRI-based assessment of meniscal injury and volume. Sixteen patients completed the week-48 assessment. Four of 18 patients had mild, self-limited adverse events, including local swelling in three high-dose patients. Compared with the low dose, the intermediate dose showed better WOMAC, AKS and Lysholm scores, whereas the high-dose group showed higher MRI-based repair rates at week 48; these exploratory findings require confirmation in controlled trials. By comparison, Okimura et al. [31] implanted particles generated from human iPSCs with BMP2, TGF-β1 and GDF5 into 1.5 mm full-thickness defects in the anterior medial meniscus of immunosuppressed rabbits. The study included nine iPSC-treated knees and three knees receiving autologous rabbit articular cartilage as a tissue-transplant comparator, and the untreated contralateral knee served as the control. After four weeks, defects with particles contained cartilage-like repair tissue with human cells still identifiable at the graft site, whereas untreated defects were only partly filled with fibrous tissue; repair was hyaline-rich in six of nine treated menisci and fibrous-cartilage-like in the remaining three. These findings illustrate two distinct approaches: iPSC-derived cartilage particles provide pre-differentiated tissue intended to engraft at the defect, whereas intra-articular IMRCs are proposed to stimulate endogenous repair through secreted mediators and immunomodulatory and matrix-regulatory activity. The IMRC trial provides early clinical safety information and exploratory outcomes but lacks a clinical control group, whereas the iPSC study provides short-term preclinical evidence of repair and of cell presence at the transplant site.

The Okimura study is best read as proof of engraftment. It was small, lasted four weeks, relied on qualitative histology without quantitative scoring, and did not assess mechanical strength or chondroprotection. It modeled a small contained defect, so it did not test root fixation or whole-meniscus function. The defects, intended as white-white, lay in the red-white zone, and human cells in immunosuppressed rabbits cannot address rejection. Whether the hyaline-rich and fibrous outcomes reflected host cues or heterogeneity of the particles was not established, and the authors note that no study has induced fibrous cartilage from iPSCs in vitro [31]. For comparison, adult synovial MSCs have been tested in rabbit, microminipig and aged primate meniscal models, including biomechanical testing in one [41,42,50,51]. The pluripotent-derived evidence rests on a single four-week iPSC study in rabbits and a phase I trial of an hESC-derived product.

Taken together, these findings show that human iPSC-derived cartilage can survive and contribute to repair in injured meniscal tissue in a small, short-term xenograft model, but they do not establish controlled generation of native meniscal fibrocartilage. No in vivo protocol has yet demonstrated reproducible generation of defined outer, transitional or inner fibrochondrocyte phenotypes. Consequently, much of the rationale for the approaches discussed below must be extrapolated from iPSC-derived articular cartilage, tendon and adult-cell meniscus studies rather than direct meniscal evidence. 

## 4. Deployment Modalities and the Emerging Biofabrication Toolkit

The critical question is how iPSC-derived cells should be delivered to a meniscal defect and organized into functional tissue. At least five deployment modalities have been proposed, at very different levels of maturity, differing in cellular maturity, architectural control, mechanical competence and translational risk (Table 2). Table 2 tags each maturity statement by evidence source. Most of the supporting evidence comes from other tissues or from non-iPSC cells, and no modality is yet rated high for biological maturity. The only direct iPSC-meniscus evidence is a single rabbit study with 4 weeks of follow-up, in which hyaline-derived iPSC cartilage particles gave hyaline-rich (6 of 9) or fibrous-rich (3 of 9) repair, without assessment of mechanics or zonal identity [31]. These categories are not mutually exclusive and are increasingly combined.

The requirements differ with the intended application, and we distinguish three. Focal-defect repair fills a contained lesion in an otherwise intact meniscus. The host roots and rim remain, so root fixation is not the main requirement, and the graft must integrate with the defect margin and be held in place. Partial replacement substitutes a resected segment. The clinical scaffolds CMI and Actifit are used with an intact peripheral rim and both horn attachments, and the Actifit studies excluded total meniscus loss and meniscal root tears [2,16,64]. They are sutured into the defect, with failure rates of 6.5% (CMI) and 18% (Actifit) at a mean follow-up of 97 and 67 months [16]. Integration with the host rim is the main difficulty. In a sheep model, a partial replacement sutured to a 2 mm rim showed no solid connection to the rim after 6 months [65]. Whole-meniscus replacement follows total or near-total meniscectomy and needs an anatomically shaped graft with functional anterior and posterior root fixation, because circumferential collagen generates hoop stress only when tethered through the roots [6]. In allograft transplantation, grafts are size-matched, fixed at the horns with soft tissue, bone plugs or a bone bridge, and placed through a mini-open or arthroscopic approach, with no technique shown to be superior [10]. 

Construct size and vascular supply scale accordingly across these scenarios. Focal-defect repair involves a contained, millimeter-scale graft relying on native perilesional vascularity and diffusion from synovial fluid [3]. Partial replacement involves a larger, centimeter-scale segment that depends on secure integration with an intact, vascularized peripheral rim [16,65]. Whole-meniscus replacement involves a full centimeter-scale, anatomically graded construct that must either achieve peripheral vascular integration of its own or tolerate prolonged avascular survival in its inner region [66,67], while bearing a native-like circumferential tensile load across its entire structure rather than at a single contained site [6]. This distinction, is based on root fixation, integration, vascular supply, construct size and mechanical demand.

### 4.1. Undifferentiated or Minimally Committed iPSCs Delivered Directly

The simplest conceivable strategy is to deliver iPSCs or early progenitors and rely on the joint environment to complete differentiation in situ. This offers manufacturing simplicity but carries a fundamental safety problem, because residual pluripotent cells can form teratomas, and the risk scales with the number of undifferentiated cells delivered [55]. To our knowledge, direct delivery of undifferentiated iPSCs to the meniscus has not been tested and remains a hypothesis. Direct delivery of undifferentiated iPSCs is therefore unlikely to be appropriate for meniscal regeneration, and the clinically relevant question is whether sufficiently lineage-restricted progenitors can retain regenerative capacity without retaining pluripotency-associated risk. This places the strategy close to the second modality.

### 4.2. Lineage-Committed iPSC Derivatives

A more controlled approach is to differentiate iPSCs before implantation into defined derivatives such as hyaline chondrocytes, iPSC-derived mesenchymal cells (iMSCs) or, ideally, meniscal fibrochondrocytes. The meniscal graft study used pre-differentiated cartilage particles [31], while iPSC-derived mesenchymal cells have been assembled into larger engineered constructs, including scaffold-free constructs implanted into osteochondral defects in immunodeficient pigs [57,68,69,70]. Pre-differentiation reduces the burden of residual pluripotency, enables release testing against defined cellular markers, and limits the amount of maturation required after implantation. The central limitation for the meniscus, however, is phenotype specification rather than differentiation efficiency. Current protocols can generate chondrogenic cells, but there is limited evidence that they reproducibly generate the type I/type II collagen-producing, zonally distinct fibrochondrocytes required for native meniscal function. 

### 4.3. iPSC Derivatives Combined with a Device or Scaffold

Because cells alone cannot initially reproduce the mechanical function of the meniscus, iPSC-derived cells can be combined with a load-bearing carrier. Candidate platforms include acellular meniscal implants already used clinically [16,17], anatomically shaped 3D-printed polycaprolactone frameworks [58], decellularized meniscal matrix [18], and fiber-reinforced hydrogel composites. Decellularized matrix provides a native extracellular environment but can lose mechanical strength and proteoglycan content during processing and remains difficult to recellularize effectively [18]. Fiber-reinforced hydrogels offer greater mechanical control: incorporation of an organized, high-porosity melt-electrowritten (MEW) microfiber network increased composite stiffness by up to 54-fold and to 405 kPa, at the lower end of the range reported for articular cartilage [71]. Stratified fiber-reinforced constructs can further impose spatial differences in mechanical and biological cues [59], while MEW provides a route to ordered fiber architectures [72]. A 3D-printed polycaprolactone scaffold with pores widening from the inner to the outer edge, seeded with rabbit bone marrow MSCs, gave more type II collagen and glycosaminoglycan on the inner side and more type I collagen on the outer side in vitro. At 24 weeks after total meniscectomy in rabbits, the neomeniscus showed fibrous outer and cartilage-like inner matrix [73]. No iPSC-derived cells were used, so this geometry-based zoning has not been tested in iPSC constructs. Meniscus-specific studies have also combined 3D-printed polymer frameworks with cell-laden or growth-factor-loaded hydrogels to promote tissue regeneration in animal models [58,60].

These mechanical gains require careful interpretation. Reinforcement can improve compressive properties, but meniscal function depends critically on circumferential tensile loading. Compressive and tensile moduli are measured under different conditions and cannot be treated as interchangeable. In the same loading mode, aligned electrospun constructs reach about 20 MPa in tension, roughly one-fifth of native circumferential values, and collagen-based constructs reach about 1 MPa (Table 3) [19,74]. Scaffolding can therefore provide a mechanical template, but it does not by itself recreate meniscal anisotropy, zonal biology or root-dependent load transfer.

### 4.4. Bioprinting with Cell-Laden Inks

Extrusion and inkjet bioprinting can position iPSC-derived cells within printable bioinks such as gelatin methacryloyl, providing spatial control over cell populations and matrix cues and, in principle, enabling different cellular phenotypes to be patterned within a single construct [58,61]. The limitation is intrinsic to most hydrogel inks: they provide cell-friendly environments but are mechanically weak and do not spontaneously reproduce the highly aligned collagen architecture of the native meniscus. Consequently, emerging approaches increasingly combine cell-laden bioinks with fiber reinforcement or MEW guidance [59,60,71]. Bioprinting with cell-laden ink is therefore best viewed as an architectural and cell-patterning technology that complements, rather than replaces, a mechanically competent scaffold.

### 4.5. Scaffold-Free Bioprinting Without Ink: The Kenzan Needle-Array Method

A fundamentally different strategy avoids exogenous biomaterials. In the Kenzan method, cell spheroids are robotically positioned onto a microneedle array and allowed to fuse before the needles are withdrawn, producing a construct composed of cells and their own extracellular matrix [63]. The absence of a permanent scaffold avoids foreign-body reactions and degradation products and produces constructs that can be handled surgically. Needle-array constructs based on adipose-derived MSCs have achieved osteochondral regeneration in rabbits [62], while scaffold-free constructs assembled from human iMSC spheroids have been implanted into osteochondral defects in immunodeficient pigs, with deposition of glycosaminoglycans and collagens in the absence of an artificial scaffold [57]. These studies establish the feasibility of building and implanting scaffold-free, iPSC-derived cartilage constructs in large-animal models, but they do not yet establish meniscal regeneration.

For the meniscus, the attraction of this approach is architectural rather than simply material. A meniscus-specific strategy has been proposed in which separately differentiated chondrocyte-like, fibroblast-like and osteoblast spheroids are spatially organized to reproduce the inner cartilage, outer fibrous tissue and bony enthesis [66]. iPSC-derived mesenchymal cells generated through a neural-crest route can form spheroid-based building blocks that fuse within days while retaining a cartilage phenotype without marked hypertrophic or fibrotic drift [70], and iPSC-derived chondrocytes have also been assembled using needle-array printing [69]. However, the gap between proof of assembly and a clinically useful meniscal graft remains substantial. Current constructs are demonstrated at cubic-millimeter rather than centimeter scale, whereas a human meniscus is a centimeter-scale, mechanically demanding tissue. Nutrient diffusion therefore becomes a major constraint, motivating the development of revascularized tissue shells and integrated microvascular units for larger engineered grafts [66,67]. Surface modification, bioadhesive strategies and suture-compatible anchors have been proposed to address fixation and integration, although none has been tested in a meniscal construct [66].

These modalities are therefore best viewed as components of an integrated manufacturing strategy rather than competing technologies. A plausible future construct could combine zone-specific iPSC-derived fibrochondrocytes, spheroid assembly or cell-laden fiber-reinforced hydrogels, mechanical maturation and an inducible safety switch [56]. MEW, fiber reinforcement, organoid assembly and genome editing have each demonstrated individual feasibility, but their integration into a mechanically competent, vascularized, surgically fixable and clinically scalable meniscus remains unresolved. Most importantly, the differentiation target that would make the construct genuinely meniscal rather than merely cartilaginous has yet to be defined and reproducibly generated.

## 5. The Central Unmet Need: Zone-Specific Fibrochondrocytes and Root-Anchored Architecture

Every deployment strategy described above ultimately depends on a cell type that current iPSC protocols have not yet reproducibly generated: the zone-specific meniscal fibrochondrocyte. Most skeletal iPSC differentiation protocols produce hyaline, SOX9-driven, type II collagen-rich chondrocytes [25,45,82]. In contrast, meniscal fibrochondrocytes co-express type I and type II collagen and are transcriptionally distinct from both articular chondrocytes and dedifferentiated monolayer meniscus cells [13].

### 5.1. Why the Differentiation Target Is Difficult

Zone-specific fibrochondrocyte is not a single terminal cell state. Single-cell profiling of healthy human meniscus identifies progenitor, proliferating, regulatory, mature fibrochondrocyte and prehypertrophic populations, with hypertrophic cells appearing in degenerated tissue [83], while matrix composition changes across development [15]. The target is therefore a spatial and developmental continuum, requiring specification of multiple related states rather than a single endpoint.

The challenge is consequently more fundamental than identifying a missing protocol. Hyaline chondrogenesis is organized around SOX9, BMP and TGF-β signaling, whereas, to our knowledge, no master transcription factor or defined signaling program has been established for meniscal fibrochondrocytes. Candidate markers identified by zonal profiling also require caution: PRRX1 and LUM are broadly expressed in mesenchymal and connective tissues, whereas ACTA2 and TAGLN increase with monolayer dedifferentiation [13], although they also mark pericyte-like cells in native outer meniscus [84]. These markers are therefore useful for defining candidate populations but are not yet sufficient for identity or release testing.

As a provisional starting point, we propose the zone-associated marker panels shown in Figure 2 to be used as a combination because no single marker is specific to the meniscus. The outer zone is represented by COL1A1, DCN and LUM with low COL2A1 and ACAN, and the inner zone by COL2A1, ACAN and SOX9 with retained COL1A1. The transcript data supporting these panels come from a small number of studies, and zonal differences vary between them. In porcine meniscus, aggrecan and type II collagen were higher in the inner region, and type I collagen, MMP2, and MMP3 were higher in the outer region in one study [85], whereas in a later study, COL1A1 was the only one of seven genes that differed in fresh tissue, being higher in the outer zone. COL2A1 and ACAN levels also vary with age and health [37]. In bulk RNA-seq, COL1A1 was also higher in the outer zone, but only 205 genes differed between zones. COL2A1 and ACAN were higher in cartilage than in outer meniscus, and LUM and PRRX1 were higher in the meniscus than in cartilage [13]. DCN and LUM were expressed across the meniscal chondrocyte population from both zones in human single-cell data [84], and COL1A1 and COL3A1 did not differ between red and white areas by immunohistochemistry [83]. Collagen- and proteoglycan-based panels therefore separate zones only partly, which is why we propose them together with the additional markers below. COL2A1, ACAN and SOX9 are also expressed by iPSC-derived chondrocytes [47], so they need to be read together with fibrous markers to distinguish fibrochondrocytes from hyaline chondrocytes. The outer-zone signal in bulk tissue is partly vascular (CD93, PLVAP, VWF, PECAM1) [13,84], which an iPSC-derived product would not carry, so these genes are not included in the outer-zone panel. The transitional zone has not been profiled separately at the cell level to our knowledge, so we describe it as an intermediate state between the two panels. Matrix-level data from porcine meniscus describe a middle zone enriched in small leucine-rich proteoglycans (FMOD, DCN, CHAD, BGN, PRELP, OGN), and MSCs on the middle-zone matrix showed intermediate COL1A1 and COL2A1 expression [86], but defining the zone at the cell level would likely need spatially resolved profiling [87]. CD49b, CD49c and CD166 were more frequent on meniscal cells than on chondrocytes in donor-matched osteoarthritic tissue and could assist sorting [88] and would be used as enrichment tools alongside the panels. In human avascular meniscus, CHAD, CILP, COMP and THBS4 were higher in tissue than in monolayer-expanded cells [89]. They are candidate readouts of native phenotype rather than zone markers, and that study did not include the vascular region.

Exclusion markers address three risks. For residual pluripotency, LIN28A has been used to estimate undifferentiated cells in iPSC-derived cartilage [55], and OCT4, NANOG and TRA-1-60 are standard pluripotency markers [34]. SOX2 is less informative because neural off-target cells in iPSC chondrogenesis also express it [47]. Detection limits depend on the method and background cell type. In one study LIN28 mRNA detected iPSCs down to 0.01% of retinal pigment epithelial cells and miR-302b in culture supernatant down to 0.001% [90], and sensitivity in iPSC-derived cartilage has not been established. These markers detect undifferentiated cells but not transformed cells, which require growth-based and in vivo assays [55]. For hypertrophy, COL10A1, ALPL, IBSP, PTH1R and IHH were low in non-hypertrophic iPSC-derived cartilage and high in MSC-derived hypertrophic cartilage [48]. IHH, MMP13, COL10A1 and ALP also increased in meniscus after ACL transection in guinea pigs and MMP13 and ALP in loaded bovine explants [91]. Even in favorable protocols, hypertrophy markers are not fully absent: collagen X was faintly detectable in some iPSC lines, and RUNX2 did not separate iPSC-derived cartilage from either articular chondrocytes or MSC-derived cartilage [48]. RUNX2 and COL10A1 also differed with cell origin among human pluripotent cell derivatives [92]. Native human meniscus contains prehypertrophic cells (MMP1, TNFAIP6, and ZIP8 mainly in the white zone) and, when degenerated, hypertrophic cells (CCL20 and EREG) [83], so thresholds would need to be set against native tissue. Type X collagen is also present in native ovine meniscus, in a subset of fibers in the white-white zone, and in fibers surrounding the root ligament fascicles [93], so type X collagen alone does not indicate hypertrophy in a meniscal construct. Cutoffs remain to be defined; a practical reference is comparison with donor articular chondrocyte and MSC-derived hypertrophic cartilage controls [48]. 

For unwanted lineages, neural (NES, PAX6, OTX2), neural crest (PAX3, FOXD3) and melanocytic (MITF) cells arise in iPSC chondrogenesis, together with ACTA2-, PRRX1- and COL1A1-positive mesenchyme resembling perichondrium [47]. ACTA2 and TAGLN increase in passaged meniscus cells [13] and also mark pericyte-like cells around vessels in the native outer meniscus [84], so they are best applied as relative thresholds against native tissue. Native reference datasets are still maturing, with small donor numbers, tissue dissociation effects, cross-species differences and loss of spatial information [87], so we expect these panels to be refined as larger spatial datasets become available. Because no single marker is unique to the meniscal fibrochondrocyte, we propose identity and release testing based on a combinatorial signature of positive panels and exclusion markers, benchmarked against native zonal references and validated with matrix and functional criteria; zone-resolved functional benchmarks do not yet exist and would need to be established.

Nevertheless, non-hyaline phenotypes can be generated from pluripotent cells. In mouse iPSCs, defined Wnt3a, activin A, TGF-β1 and bFGF exposure produces PRG4-positive superficial-zone and fibroblast-like cells [94], while staged modeling of skeletal development provides a framework for separating stable cartilage from hypertrophic and bone-fated states [46]. Standard chondrogenic routes are also increasingly reproducible and well characterized [36,82]. Patterned assembly of regionally distinct cartilaginous organoids has produced zone-specific functionality in vivo, providing a precedent for building a graded tissue from pre-specified cellular units rather than relying on positional cues alone [95]. WNT signaling modulation that currently serves to limit off-target and hypertrophic differentiation may therefore also provide a route toward fibrochondrocyte specification [47,48].

### 5.2. Developmental Origin and Under-Used Specification Levers

Developmental biology provides a more rational starting point than empirical optimization of chondrogenic culture conditions. Lineage tracing in mice shows that the meniscus, cruciate ligaments, synovial lining and articular cartilage arise from Gdf5-expressing joint interzone progenitors rather than the surrounding cartilage anlagen [96]. Temporal lineage tracing further demonstrates continuous recruitment into the interzone, with different joint tissues labeled according to developmental timing [97]. This is developmental evidence from animal models. From it, the meniscal progenitor can be viewed as a temporally specified interzone-derived state rather than as a conventional chondrocyte that subsequently acquires fibrous characteristics, but this interpretation has not been tested in human cells.

The proposed route from pluripotent cells to meniscal fibrochondrocytes (Figure 2) combines three levels of evidence, which we separate here. First, the GDF5-positive interzone origin of the meniscus is supported by developmental evidence from animal lineage tracing [96,97]. Second, some individual steps have been shown in human pluripotent cells, but not toward the meniscus. Paraxial mesoderm derived from human pluripotent cells gave rise to SOX9-positive chondroprogenitors and then to GDF5-positive cells that formed largely unmineralized, PRG4-expressing permanent-type cartilage after ectopic transplantation. Transcriptomic analysis suggested that these GDF5-positive cells have teno/ligamentogenic potential, and they expressed SCX under some culture conditions [92]. Tendon and ligament programs can also be induced in iPSC-derived mesenchymal cells through MKX or SCX together with mechanical loading [98,99]. Third, the steps that connect these pieces are hypotheses. The GDF5-positive cells correlated only weakly with mouse interzone cells and more closely with tendon and ligament precursors [92], so an interzone identity has not been shown, and the reported iPSC meniscal graft study generated hyaline cartilage particles directly, without such an intermediate [31]. A GDF5-positive intermediate is therefore a plausible branching point for meniscal specification, but it remains an experimental hypothesis.

A second underused lever is the tenogenic component of fibrochondrocyte identity. Whereas most iPSC protocols focus on type II collagen production, tendon and ligament programs can be induced in iPSC-derived mesenchymal cells through MKX or SCX together with mechanical loading [98,99]. This evidence comes from tendon models, not from the meniscus. In one of these studies, SOX9 expression also rose in SCX-overexpressing cells stretched for 7 days [98]. SCX and SOX9 are co-expressed in mouse progenitors that form the attachment sites of tendons and ligaments to cartilage and bone [100,101]. In the human pluripotent-cell study of GDF5-positive cells, however, SOX9 was largely absent from the GDF5-positive cells in culture and rose again in chondrogenic pellet culture [92], so a state in which GDF5, SCX and SOX9 are active together has not been shown. Titrated activation of chondrogenic (SOX9) and tenogenic (SCX) programs within a GDF5-positive intermediate is therefore a hypothesis for generating cells with the type I/type II collagen profile of meniscal fibrochondrocytes. This combination has not, to our knowledge, been tested for meniscal regeneration; the SCX and SOX9 levels that would define each zone are unknown, and no master transcription factor has been established for meniscal fibrochondrocytes. In summary, the GDF5 origin is supported by developmental evidence, and individual induction steps by human pluripotent-cell and tendon-model data, and the combined GDF5–SCX–SOX9 route is a hypothesis for experimental testing.

Oxygen tension provides a complementary, non-genetic zonal cue. The inner meniscus is avascular and hypoxic, whereas the outer region is vascularized, and the two populations respond differently to reduced oxygen. At 5% oxygen, outer-zone cells increased SOX9 and type II collagen while reducing type I collagen, whereas inner-zone cells increased both type I and type II collagen without a corresponding increase in SOX9. HIF-1α and downstream targets were also higher in fresh inner than outer meniscus tissue [102]. Although this evidence derives from a single aggregate-culture study and has not been tested in a graded construct, oxygen tension, together with substrate mechanics and YAP regulation [45], offers a plausible means of reinforcing zonal identity after developmental specification. These findings support a developmental, staged approach rather than simple optimization of conventional chondrogenic cues.

### 5.3. Is Zonal Identity Intrinsic or Positional?

Whether zonal identity can be programmed intrinsically is critical to the feasibility of iPSC-based regeneration. Developmental studies favor a substantial intrinsic component: regional differences in matrix composition and cell fate are present at birth, before continuous weight-bearing, and become further specialized during growth [103]. Reducing neonatal hindlimb loading did not substantially alter meniscal morphology, Col1a1 or Col2a1 reporter populations, or nanoindentation micromechanics, despite marked effects on gait and other musculoskeletal tissues. These findings could be interpreted conservatively because sciatic nerve resection does not eliminate loading and also alters neurotrophic input and contralateral compensation [104]. Nevertheless, they suggest that meniscal zonation is established developmentally and is not generated by mechanical loading alone. This supports programming zonal identity before implantation rather than expecting an initially non-specific hyaline graft to acquire appropriate regional phenotypes from its location within the joint.

These zonal reference profiles are not fixed targets, because native values vary with donor age, degeneration and anatomical location, and the available datasets do not separate these factors well. The developing meniscus is highly cellular and vascular, and by adulthood only the peripheral 10 to 30% remains vascularized [35]. In joints with no or minimal osteoarthritis, aging was associated with increased Safranin-O staining, lower cell density, acellular zones and some loss of fiber organization, with the earliest changes along the inner rim [105]. In the same cohort, damage also differed between locations: in knees with tears, the posterior horn was involved in 66% and the anterior horn in 11%, and the medial meniscus was damaged more often than the lateral (28% vs 12%) [5]. In osteoarthritic menisci, the posterior horns were the most affected and the anterior horns the least [105]. In lateral menisci from knee replacement patients, however, the intermediate and anterior regions were more often highly degenerated than the posterior region, and the equilibrium compressive modulus was higher in the anterior region than in the intermediate and posterior regions [106]. The reported effects of degeneration on mechanical properties also differ between studies. Compressive moduli were lower in more degenerated menisci, by 35 to 72% in three of four regions in one study [107] and in equilibrium modulus in another [106], and the tensile modulus did not change significantly in either. In contrast, indentation moduli were higher in severely than in mildly degenerated joints [108]. In that study the severely degenerated joints came from donors with a mean age of 82 years and the mildly degenerated joints from donors with a mean age of 48 years, so degeneration and age were confounded [108]. Several of the datasets used in this review also come from aged or osteoarthritic tissue. The micro-CT vascularity data were obtained from six cadaveric menisci of three male donors with a mean age of 75 years [14], and the regional cell-phenotype data [88] came from total-knee-replacement donors aged 46 to 87 years (mean 66 years). For regeneration, this means that a construct for a young patient is best compared with young, non-degenerate tissue from the same region, rather than with values from aged or osteoarthritic tissue. Because values from degenerate tissue can be lower or higher depending on the grading system and test method and are confounded with age, they are better read as provisional than as design targets. Reference profiles are most useful when they report the donor age, degeneration grade and grading system, anatomical region and test method they came from.

### 5.4. Architecture: Alignment and Root Fixation

Even a correctly specified fibrochondrocyte is insufficient without the architecture that converts cellular and matrix properties into meniscal mechanics (Figure 3). The human meniscus has a circumferential tensile modulus of 58–152 MPa across regions and layers (regional means 90–102 MPa) in quasi-static tests at 5%/min and a radial tensile modulus of 2–23 MPa [75]. Compressive properties depend strongly on loading rate: the unconfined modulus at 12% strain is 76–83 kPa at equilibrium and 605–718 kPa at a physiological strain rate [78], and the confined aggregate modulus is 0.11 ± 0.08 MPa [79]. Because these values vary with test configuration, hydration, region, species and age, each construct value in Table 3 is paired with its test conditions. Aligned electrospun PCL seeded with bovine meniscal cells reached 19.7 MPa in tension after 10 weeks [19], about one-fifth of the human regional means, and clamped collagen constructs reached about 1 MPa [74]. Fiber-reinforced hydrogels reached up to 405 kPa in unconfined compression [71], at the lower end of the reported range for articular cartilage. The main deficit is therefore in tension and directionality rather than compression. No iPSC-derived meniscal construct has reported mechanical data.

Root anchorage is equally fundamental. Circumferential collagen bundles generate hoop stress only when tethered through the anterior and posterior roots and their bony entheses [6]. Root tears, which include avulsions and radial tears adjacent to the root, impair hoop-stress transmission, cause extrusion and are biomechanically similar to total meniscectomy [6]. In a cadaveric study of the lateral meniscus, posterior root avulsion or an adjacent radial tear decreased contact area and increased mean and peak contact pressure, and in situ pull-out suture repair improved contact pressures [7]. A whole-meniscus construct that reproduces internal fiber alignment but lacks functional root fixation therefore cannot restore native meniscal mechanics. These root requirements apply to whole-meniscus replacement, whereas focal-defect repair and partial replacement rely on intact host roots.

Fiber-aligned and melt-electrowritten scaffolds, anatomically printed frameworks and anisotropic functionalized constructs can impose matrix alignment and guide cellular organization [19,59,71,109,110]. Scaffold-free assembly and spheroid fusion can generate cohesive tissue and permit progressive proteomic and mechanical maturation [20,57,111], while mechanical conditioning can promote hierarchical collagen organization [74]. These approaches address architecture, but their success ultimately depends on an unambiguous cellular target. Because meniscal phenotype varies by zone and is altered during cell isolation and culture [13,83,89], the candidate marker panels proposed above would need functional validation against native zonal references before iPSC product development.

### 5.5. Vascular Supply and Nutrient Transport

Clinical-scale regeneration introduces a second constraint that small animal defects largely avoid: nutrient transport across centimeter-scale tissue. The native meniscus is vascularized primarily in its outer third, whereas the inner region is avascular and relies on diffusion from synovial fluid, aided by cyclic loading [3]. A graft must therefore remain viable before peripheral vascular integration while maintaining appropriate cell density, thickness and matrix permeability. Current scaffold-free constructs remain at approximately cubic-millimeter scale, whereas the human meniscus is centimetric; prevascularized tissue shells and integrated microvascular units are being explored to address this scaling problem [66,67].

Native tissue values and construct data give a quantitative frame for this constraint. In lateral menisci from six women aged 48 to 65 years undergoing knee replacement, cell density was about 27,200 cells/mm^3^ in the vascular region and about 12,800 cells/mm^3^ in the avascular region, and avascular regions lay more than 5 mm from the outer periphery [112]. A computational model of the human meniscus predicted that oxygen stays at the synovial fluid level throughout the tissue, that glucose falls to 4.2 mM at its lowest point, that removing the vessels lowers glucose to as little as 55% of the vascularized baseline, and that shrinking the vascularized zone from 30% to 10% of the tissue lowers it by 5% or less. Quantitative data on meniscal fibrochondrocyte metabolism were sparse, so the model used values from other cartilaginous tissues, namely intervertebral disk, articular cartilage and temporomandibular joint [113].

Engineered constructs are more demanding. In polymer scaffolds seeded with bovine chondrocytes (4 mm diameter, cored from 5 mm blocks), oxygen at the center fell to as low as 2% after 14 and 27 days of culture, measured with the constructs bathed in air-saturated medium, and the polymer’s oxygen diffusion coefficient was about 25 times lower than that of cartilage. The model in that study was most sensitive to cell density, oxygen consumption per cell and the diffusion coefficient [114]. Isolated bovine articular chondrocytes consumed oxygen at a basal 9.6 × 10^−16^ mol/cell/h, rising to less than 18.4 × 10^−16^ mol/cell/h in 1.3 mM glucose, and in 2 mm thick agarose constructs seeded at 40 × 10^6^ cells/mL (40,000 cells/mm^3^, about three times the avascular meniscus value above) oxygen reached 5 µM in low-glucose medium and 57 µM in high-glucose medium [115]. In human meniscus fibrochondrocytes seeded at 5 × 10^6^ cells/cm^3^ (5000 cells/mm^3^) on collagen scaffolds 6 mm in diameter and 3.5 mm high, a core with less matrix and fewer cells formed by 3 weeks and persisted through a further 3 weeks of culture in all groups and donors [116].

These data identify the parameters available for a graft: thickness, seeded cell density, oxygen regime, medium glucose and convective supply. Gentle dynamic compression, forced perfusion during pre-culture and constructs with medium channels have been proposed for matrix-poor cores [116] and have not been tested in iPSC-derived meniscal constructs. The oxygen regime also acts on phenotype. In human meniscus fibrochondrocyte constructs, 3% oxygen with dynamic compression lowered COL10A1 about 9-fold relative to static culture in 20% oxygen [116], and 3% oxygen suppressed the hypertrophic phenotype over 9 weeks, but mechanical properties rose only in 20% oxygen, which reached one-fifth of the native instantaneous compression modulus with an increasingly hypertrophic phenotype [117]. Five days at 3% oxygen also raised VEGF expression relative to 20% oxygen [116], so VEGF could serve as a readout when an oxygen regime is set for an inner-zone graft. Vascular supply does not need to reach the inner zone of an implanted graft. In the same model, shrinking the vascularized zone to 10% of the tissue changed glucose by 5% or less, which suggests a small vascularized rim could be enough [113], and a recent review notes that excessive vascularization of the inner meniscus may disrupt its native physiology, so vascular cues need to be spatially controlled [118]. Prevascularized shells and microvascular units developed for other grafts could therefore be confined to the periphery, while the inner region relies on synovial fluid and cyclic loading, as in native tissue.

Vascularization is therefore part of the target tissue phenotype itself, not only a delivery problem. The outer vascular zone supports peripheral integration and remodeling, whereas the inner avascular environment contributes to its distinct oxygen and cellular state [102]. A uniformly vascularized or uniformly avascular construct would reproduce neither region. Vascular architecture could therefore be designed alongside cellular identity and matrix organization and remains one of the least developed components of a clinically relevant iPSC-derived meniscus.

## 6. Translational Barriers, Preclinical Validation and Clinical Trials

An iPSC-derived meniscal product must clear the barriers common to all pluripotent stem cell therapies. Tumorigenicity is a primary safety concern, driven principally by residual undifferentiated cells and incomplete lineage restriction. Accordingly, release strategies could combine sensitive assays for residual pluripotent cells with in vivo tumorigenicity testing appropriate to the intended cell product [55], while engineered safeguards such as inducible suicide genes and cell-cycle control provide additional layers of protection [56]. Genomic stability is a related and less frequently discussed requirement: clinical cell banks must be characterized for chromosomal abnormalities and acquired genomic alterations before and after the expansion and differentiation steps required for manufacturing, and karyotype and pluripotency verification are already incorporated into the qualification of edited clinical cell candidates [119]. Immunogenicity also requires careful consideration. iPSC-derived cartilage exhibits relatively low expression of major histocompatibility complex molecules under basal conditions, but interferon-γ can increase MHC class I expression, indicating that its apparently low immunogenicity may change under inflammatory conditions [120]. This distinction is particularly relevant to a meniscal graft, because the outer meniscus is vascularized and therefore more exposed to immune surveillance than avascular cartilage. For allogeneic products, potential strategies include HLA-matched banking or engineering hypoimmunogenic cell lines, with targeted HLA modification providing one route toward broader donor compatibility [119]. Editing is not a complete solution: loss of MHC class I expression from monkey iPSC-derived cartilage reduced conventional recognition but was associated with recruitment of natural killer cells and other leukocytes, illustrating how eliminating one recognition pathway can expose another [121].

Phenotypic instability represents a second major translational barrier and is particularly important for meniscal regeneration. Hypertrophic differentiation can compromise both safety and function because type X collagen-expressing cells can promote mineralization and endochondral ossification; consequently, stable suppression of hypertrophic programs must be demonstrated during differentiation, maturation and after implantation [47,48,49]. More specific to the meniscus is the challenge of maintaining the distinction between fibrous, fibrocartilaginous and hyaline-like phenotypes [13,31]. A related concern is that phenotype stability has generally been evaluated under relatively controlled culture conditions, whereas a clinical meniscal graft would experience cyclic mechanical loading and, in many patients, an inflammatory synovial environment. iPSCs derived from healthy and osteoarthritic donors can differ in chondrogenic behavior and epigenetic state [53], raising the possibility that donor state could influence reproducibility in autologous approaches. Early qualification should therefore test candidate cells under clinically relevant inflammatory conditions, including exposure to interleukin-1β, tumor necrosis factor-α, and, where feasible, patient-derived synovial fluid, while assessing maintenance of the intended meniscal phenotype and matrix program. Such stress testing would provide a more stringent assessment of phenotypic stability than conventional culture alone.

Good Manufacturing Practice, batch consistency, potency assays and scale-up remain substantial challenges, although several enabling technologies now demonstrate that large-scale production of pluripotent-derived mesenchymal and chondrogenic cells is technically feasible. Stirred-bioreactor culture of iPSC-derived limb-bud mesenchymal cells generated cartilaginous tissue from an input of approximately one million cells, supporting a route to scale through controlled bioreactor culture [122]. Automated manufacturing platforms have also been developed to integrate iPSC expansion, mesenchymal and chondrogenic differentiation, and downstream processing under defined quality-control conditions [123]. Clinical experience with iPSC-derived products provides an additional proof of manufacturing feasibility. The first completed clinical trial of an iPSC-derived cell product used CYP-001, an iPSC-derived mesenchymal stem cell product, in patients with steroid-resistant acute graft-versus-host disease, demonstrating the feasibility of GMP-compliant manufacture and clinical dosing of a pluripotent-derived cell therapy [124]. A 14-day monolayer plus pellet protocol for iPSC-derived chondroprogenitors was reproduced in two further iPSC lines and at a second site, but one of the three lines did not proliferate in defined serum-free medium [125]. However, the manufacturing requirements for a living, structurally organized meniscal graft are substantially greater than those for an injectable cell product. For meniscal regeneration, potency therefore cannot be defined solely by viability, surface markers or generic chondrogenic gene expression. A clinically meaningful potency framework should link cellular identity to functional outputs, including matrix synthesis, collagen organization, mechanical maturation and maintenance of the intended zone-specific phenotype. Manufacturing costs for a structurally organized construct will substantially exceed those of injectable cell products or acellular scaffolds, and cost-effectiveness will need dedicated evaluation before broad adoption [126,127]. 

Preclinical validation would consequently need to progress from cellular safety and tissue formation to whole-construct mechanical and functional performance. Existing models span foundational iPSC chondrogenesis, safety engineering, scaffold-free and device-based construction, and the direct meniscal engraftment study described above (Table 4). Large-animal meniscal models, including ovine constructs incorporating spatiotemporal growth-factor delivery, provide platforms for evaluating tissue formation and mechanical function [128], while scaffold-free needle-array cartilage constructs have progressed to porcine models [57]. These studies provide relevant platforms for testing construct size, fixation, integration, load transfer and chondroprotection, but none yet reproduces the full combination of zone-specific meniscal phenotype, circumferential tensile function and root-dependent mechanics required for a clinically relevant graft. Preclinical programs should therefore progress through a staged framework, beginning with biodistribution and tumorigenicity, followed by small-animal proof of concept and then large-animal assessment of mechanical competence, meniscal extrusion, root integration, durability and protection of the articular cartilage over clinically meaningful follow-up periods.

Clinical translation of iPSC-derived cartilage has begun, but the available evidence remains limited and is centered primarily on articular cartilage rather than meniscal tissue replacement. 

In Japan, the TACK-iPS study evaluated allogeneic iPSC-derived cartilage for knee articular cartilage defects. Four patients underwent transplantation; one was discontinued after an adverse event unrelated to the study, and three completed 12-month follow-up. The jRCT registry, last updated January 2024, reports outcome data: no tumor formation on CT, MRI, or whole-body CT in any patient; successful cartilage integration on 3D-MOCART MRI in all four at 6 months; arthroscopic ICRS grade improvement in all three followed patients at 12 months (two from grade 3 to grade 2, one from grade 3 to grade 1); biopsy-confirmed hyaline or hyaline/fibrocartilage tissue in all three; and improvement across all five KOOS subscales from baseline to one year, including pain (67 to 89) and quality of life (41 to 58) (jRCT registry, jRCTa050190104 [29]). These are registry-posted results, not yet a peer-reviewed publication, from a small, single-arm, uncontrolled study.

This establishes clinical experience with administration of an iPSC-derived cartilage product but does not establish efficacy of structural cartilage regeneration. A human embryonic stem cell-derived immunity-and-matrix-regulatory cell product has also completed a phase I trial for meniscal injury and was reported to be safe over 12 months, but this approach was delivered as an intra-articular cell therapy rather than a tissue-replacing meniscal graft [30]. Table 5 lists these studies and the established meniscal products. 

The translational pathway for an iPSC-derived meniscal graft therefore requires more than demonstration of cartilage formation or short-term cell survival. Before first-in-human testing, the product would need to demonstrate reproducible zone-specific cellular identity, genomic and tumorigenic safety, manufacturing consistency, functional potency, mechanical competence, stable integration with host tissue and fixation sites, and durable preservation of articular cartilage. Table 6 summarizes how focal-defect repair, partial replacement and whole-meniscus replacement differ in root fixation, integration and delivery. The iPSC evidence to date comes from a small contained-defect model and does not address root fixation or whole-meniscus function [31]. Milestones, therefore, differ by indication: focal-defect repair depends on integration with the defect margin, partial replacement on integration with the host rim, and whole-meniscus replacement additionally on functional root attachment.

Assays with defined sensitivity exist for the main safety attributes, although the reported limits come from other products and cell backgrounds. For residual undifferentiated cells, one-step RT-ddPCR for the transcripts ESRG, LINC00678 and LIN28A detected iPSCs spiked into iPSC-derived cardiomyocytes down to 0.0003% in a multisite evaluation [130]. The highly efficient colony-formation assay used for iPSC-derived cartilage has a reported limit of detection of 0.001%, improved to 0.0002% with a sorting step, although it takes about one week and needs a large sample [90]. For transformed cells, a growth-based spike test in iPSC-derived cartilage found that 100 HeLa cells mixed into 150 mg of tissue accelerated cell growth, whereas unspiked tissue reached growth arrest and senescence [55]. In vivo, the 50% tumor-producing dose of HeLa cells was 1.3 × 10^4^ cells in NOG mice compared with 4.0 × 10^5^ cells in nude mice, about 30-fold lower, and embedding the cells in Matrigel lowered it further [131]. For genomic stability, karyotype and pluripotency verification are already incorporated into the qualification of edited clinical cell candidates, as noted above [119], although a numerical acceptance limit is not established. For hypertrophy, COL10A1, ALPL, IBSP, PTH1R and IHH have been used to separate non-hypertrophic from hypertrophic cartilage, without a defined numerical cutoff [48]. Genomic stability would need a defined resolution, with higher-resolution copy-number or sequencing methods considered alongside karyotyping, and hypertrophy thresholds would need to be set against donor-matched native meniscus rather than an arbitrary cutoff.

None of these limits was measured in an iPSC-derived meniscal product, and detection limits depend on the background cell type: in the ddPCR study, the full spike-and-recover process varied between three laboratories with coefficients of variation of about 32 to 57%, driven mainly by the spiking and sample preparation steps rather than the assay itself [130]. Each assay therefore needs qualification in the final product matrix, spiking known numbers of iPSCs and surrogate transformed cells into iPSC-derived meniscal cells and graft material, and establishing specificity, limit of detection, precision and recovery in that matrix. A validated potency assay for an iPSC-derived meniscal graft is not available from the studies reviewed here. We propose a matrix-functional assay linking cellular identity to function: the zone marker panel described above, glycosaminoglycan and collagen content, collagen fiber alignment, and tensile modulus measured against the targets in Table 3 after a period of cyclic loading, with hypertrophy markers read against donor-matched native tissue. Acceptance limits are not yet defined and would need to be set against the native meniscus and validated for specificity, precision and reproducibility before use as a release test.

The first clinical evaluation should prioritize safety over efficacy. The first-in-human transplantation of an iPSC-derived cell product set this precedent: a single patient received an autologous iPSC-derived retinal pigment epithelium sheet, with safety and the adverse-event profile as the primary outcome, and a second enrolled patient was never treated after genomic copy-number aberrations were detected in that patient’s iPSC line during safety qualification [132]. A comparable staged approach would be appropriate for a first iPSC-derived meniscal graft: a small, open-label, dose- or size-escalation design with pre-specified stopping rules, using tumorigenicity, immunogenicity and graft-integrity monitoring as primary endpoints, following the precedent of the TACK-iPS cartilage study [29] and the hESC-IMRC meniscal trial [30]. Neither of these existing clinical experiences included a concurrent comparator group [29,30]. Patient selection will also determine whether this or any later trial can be interpreted. Experience with meniscal allograft transplantation shows that outcomes are influenced by limb alignment, ligamentous stability and articular cartilage condition; patients with uncorrected malalignment, instability or advanced cartilage degeneration are generally excluded [10]. A first iPSC-derived graft trial could define these factors prospectively as prespecified covariates, otherwise any chondroprotective effect could be confounded by the mechanical environment.

A single-arm safety trial cannot support efficacy claims. A subsequent trial would need a concurrent comparator group, since an untreated or sham control is not appropriate for symptomatic meniscal injury; depending on indication, an active comparator such as meniscal allograft transplantation [11] for whole-meniscus replacement or the current standard repair technique for focal-defect indications would allow interpretable comparisons, with randomization once safety is established. Symptomatic improvement, structural regeneration and chondroprotection are distinct outcomes and should not be conflated; trial design frameworks for cartilage and joint repair increasingly recommend matching the primary outcome to the therapeutic target and disease stage rather than defaulting to patient-reported outcomes alone [133]. Patient-reported outcomes (KOOS, IKDC and visual analog pain scores) capture symptomatic improvement and can be assessed within the first one to two years. Structural regeneration, meaning meniscal morphology, volume and integration on standardized imaging, requires the graft to have matured and integrated before it is informative. Chondroprotection is the most demanding outcome: symptomatic relief or preserved meniscal structure does not by itself demonstrate that the articular cartilage has been protected. Quantitative cartilage MRI (T2 and T1rho mapping) and, over the longer term, joint-space narrowing or other radiographic measures of osteoarthritis progression would be needed to support a chondroprotective claim, with follow-up extending over years rather than months.

Any translational program should ensure patients are informed of current limitations. Reproducible generation of zone-specific meniscal fibrochondrocytes has not yet been demonstrated, and long-term mechanical durability and chondroprotection remain unproven. An allogeneic off-the-shelf strategy, whether through HLA-matched banks or hypoimmunogenic edited lines [119], would reduce the manufacturing interval and improve access but introduces immunological considerations requiring prospective monitoring. The success of an iPSC-derived meniscal graft will ultimately be judged by whether it restores a functional knee, not simply whether cells survive or a defect becomes filled.

## 7. Regulatory Landscape

Regulatory strategy cannot be separated from translation. For an iPSC-derived meniscal graft, the regulatory pathway will depend not only on the cellular component but also on the function and regulatory status of the scaffold, manufacturing process and any genetic modifications. In the USA, iPSC-derived therapeutic products are generally regulated as biological products, while cell-containing tissue-engineered constructs may also raise combination-product considerations when a biologic and device constituent are combined to achieve the intended therapeutic effect. FDA guidance recognizes regenerative medicine products incorporating structural scaffolds, making early determination of product classification and the respective regulatory responsibilities important for development, including the FDA’s framework for premarket pathways for combination products [134]. The Regenerative Medicine Advanced Therapy (RMAT) designation, established under Section 3033 of the 21st Century Cures Act, provides opportunities for intensive regulatory interaction and expedited development for eligible regenerative medicine therapies addressing serious conditions and unmet medical needs [135]. 

In the European Union, iPSC-derived cell and tissue-engineered products fall within the advanced therapy medicinal product (ATMP) framework under Regulation (EC) No 1394/2007 [136]. A product combining a structurally functional scaffold with an iPSC-derived cellular component may be classified as a combined ATMP, assessed through the Committee for Advanced Therapies’ Article 17 procedure [137], with the regulatory package required to address the complete product: cellular identity, potency and safety, together with scaffold performance, degradation and integration.

Japan’s conditional and time-limited approval pathway for regenerative medical products is established under the Act on the Safety of Regenerative Medicine (Act No. 85 of 2013) [138]. This pathway’s first test illustrates that conditional approval does not remove the requirement to demonstrate efficacy: HeartSheet^®^, an autologous skeletal-myoblast-derived cell sheet, received conditional and time-limited approval from Japan’s Pharmaceuticals and Medical Devices Agency (PMDA) in September 2015, but in July 2024 PMDA’s review determined that efficacy had not been demonstrated and full approval was not granted [139], the first such denial under the scheme. South Korea has established a dedicated regulatory track for cell-based regenerative products under the Act on the Safety of and Support for Advanced Regenerative Medicine and Advanced Biological Products (Act No. 17472, 2020) [140], combining expedited review with post-marketing evidence requirements [141]. China has likewise developed dedicated regulatory tracks for cell-based regenerative products, combining expedited or conditional review with post-marketing evidence requirements [142]. For an iPSC-derived meniscal graft intended for international translation, these frameworks present additional points of early regulatory alignment beyond those discussed above.

For an iPSC-derived meniscal graft, this experience underscores the importance of defining clinically meaningful efficacy endpoints before approval rather than relying on structural repair or surrogate measures alone. A product intended to restore meniscal function would ultimately need evidence linking zone-specific tissue formation to patient benefit, mechanical competence, durable integration and preservation of the articular cartilage. 

The regulatory implication is therefore broader than product classification. Product development could begin with a predefined control strategy covering cell identity, residual pluripotency, genomic stability, tumorigenicity, potency, scaffold properties, manufacturing consistency and release testing. This is particularly important for iPSC-derived products, for which variability in differentiation, genetic and epigenetic abnormalities, immunogenicity, tumorigenicity and GMP-scale manufacturing remain recognized translational challenges [143]. For the final meniscal graft, mechanical performance, fixation, degradation or remodeling behavior, host integration and long-term joint protection should therefore be evaluated as properties of the complete therapeutic product. Early regulatory consultation should consequently occur before pivotal preclinical studies so that the assays and animal models used to establish safety, potency and function are aligned with the evidence ultimately required for first-in-human and marketing applications.

## 8. Limitations of the Evidence 

The case for pluripotent sources should be weighed against several plausible alternatives. Adult cell sources may remain preferable for some meniscal indications because they avoid pluripotency-associated safety risks and may require less extensive manufacturing. Synovial mesenchymal stem cells have improved meniscal healing after repair in microminipigs [50] and promoted repair in a porcine meniscus injury model [51]. Uncultured adipose-derived regenerative cells have likewise promoted meniscal tissue formation after partial meniscectomy in rats without prolonged ex vivo expansion [40]. These approaches nevertheless have limitations, including heterogeneity of the starting cell population, donor-to-donor variability and less precise control over lineage specification.

Acellular and synthetic devices, discussed above, represent another competing pathway. Scaffolds seeded with chondrocytes have also been evaluated in a sheep total meniscectomy model, where cell seeding increased cartilage formation numerically but did not reach statistical significance [144]. 

An iPSC-derived meniscal product would therefore need to demonstrate a reproducible biological or functional advantage sufficient to justify its greater manufacturing complexity over adult-cell and scaffold-based alternatives. Several limitations of the current evidence could be stated plainly. Direct meniscus-directed iPSC evidence remains sparse and is dominated by a single short-duration rabbit study. Conclusions about iPSC-based meniscal regeneration therefore depend substantially on extrapolation from articular cartilage, tendon and adult-cell meniscus studies. No validated protocol currently generates a reproducible panel of outer, transitional and inner meniscal fibrochondrocytes, and no consensus phenotype or potency assay exists for such a product [13,83]. Most iPSC skeletal studies continue to focus on hyaline cartilage, which cannot be assumed to reproduce the graded fibrocartilage of the native meniscus [27].

The evidence base also has important methodological limitations. Biomechanical benchmarking against native meniscus is heterogeneous, with substantial variation in specimen preparation, loading mode, anatomical region and reported outcome measures [75,77,145]. Several foundational datasets are decades old, while engineered constructs are often tested under conditions that do not reproduce the combined circumferential tensile, compressive and shear loading experienced in vivo. Animal models are similarly heterogeneous, with substantial variation in lesion type, species, surgical technique and outcome assessment. Standardized large-animal studies that simultaneously assess tissue formation, mechanical function, extrusion, integration and protection of articular cartilage remain limited. Long-term safety data for a mechanically loaded, vascularized meniscal graft in an immunocompetent large-animal environment are also unavailable, particularly for tumorigenicity, hypertrophic drift and immunogenicity [47,55,56]. The resulting evidence base does not currently establish either superiority over existing approaches or durable clinical benefit. As a narrative review, the selection of studies reflects the judgment of the authors and is intended to identify principal translational uncertainties rather than provide a systematic comparison of competing technologies.

Several failure modes could defeat the program outlined above. First, the intended zonal phenotype may not remain stable after implantation. Meniscal cell identity is influenced by extracellular matrix, mechanical loading, oxygen tension and developmental context. A protocol that generates zone-associated markers in vitro may therefore fail to maintain the corresponding phenotype under the mechanical and inflammatory conditions of the joint. Second, inappropriate differentiation may compromise both function and safety. iPSC-derived chondrogenic cells may undergo hypertrophy, mineralization or endochondral ossification after implantation despite apparently stable differentiation in vitro [47,49]. Third, structural failure may occur even when cellular differentiation is successful. Meniscal function depends on circumferential tensile load transfer and secure root attachment (Table 3) rather than on compressive properties alone. No current biofabrication strategy has demonstrated all of these features simultaneously at whole-meniscus scale. Fourth, scaling may introduce biological and engineering failures that are not apparent in small constructs. A human meniscus is a centimeter-scale tissue with a spatially organized matrix and heterogeneous vascularity. Maintaining cell viability, matrix maturation, nutrient delivery and zonal organization during scale-up remains unproven. Finally, successful implantation requires durable integration rather than tissue formation alone. A graft must integrate with the surrounding meniscal tissue and native root attachments, remain mechanically competent under repetitive loading, and remain free of residual pluripotent or otherwise inappropriate cell populations over the long term. These risks are interdependent: inadequate vascular supply may impair maturation, poor fixation may increase graft loading, mechanical or inflammatory cues may alter phenotype, and incomplete lineage restriction may create delayed safety risks. They could therefore be tested as interacting failure modes throughout development rather than treated as independent end-stage checks.

## 9. A Sequential and Testable Roadmap

The translational path could be structured around sequential, testable milestones (Figure 4). The starting point is to define the target product by establishing reproducible molecular and functional signatures for inner, transitional and outer meniscal fibrochondrocytes, informed by native single-cell and bulk transcriptomic datasets and validated against matrix composition and mechanical behavior [13,83]. With this target defined, iPSCs could be directed through developmentally appropriate, interzone-like progenitor states [92,96] toward these zone-specific phenotypes, rather than toward a generic hyaline chondrocyte fate. Candidate cells could in turn be challenged under meniscus-relevant mechanical, inflammatory and oxygen conditions to determine whether their identity and function are stable before they are incorporated into a graft. Validated cell populations could subsequently be integrated into a construct that reproduces the essential architecture of the native meniscus, including circumferential collagen alignment, zonal organization and a mechanically competent root interface [6,7,57,58,59,71,72]. Dynamic maturation could determine whether the intended matrix architecture and mechanical properties develop and remain stable [111]. The construct would also need to demonstrate that these properties can be maintained at clinically relevant dimensions, with nutrient and vascular strategies appropriate to the graded biology of the tissue [66,67]. The complete product must then undergo integrated safety, potency, manufacturing and regulatory qualification as described above [55,56,146,147]. The final translational test is functional rather than histological. Large-animal studies could then determine whether the construct can survive implantation, remain integrated and mechanically competent, maintain root fixation, resist extrusion and protect the articular cartilage under clinically relevant loading [57,128]. A meaningful development milestone would be a zonally graded iPSC-derived meniscal construct with defined cellular identities, controlled anisotropy, functional root attachment, reproducible mechanical properties and an acceptable safety profile. Milestones differ by indication: focal-defect repair depends on integration with the defect margin, partial replacement on integration with the host rim, and whole-meniscus replacement additionally on functional root attachment. Each stage should have predefined, measurable go/no-go criteria. The numerical values and time periods below are proposed development examples informed by published studies, not validated or universally applicable release criteria for an iPSC-derived meniscal product; each would require justification and qualification for the intended product, assay and clinical indication. Examples include the following: (1) cell-source safety, confirmed genomic stability and residual undifferentiated or transformed cells assessed with qualified assays carrying a documented limit of detection in the final product matrix (published limits, such as ≤0.0001% by digital soft-agar colony formation [148] or ≤0.001% by droplet digital PCR [149], may inform assay development but are not validated release thresholds for this product); (2) fibrochondrocyte identity, reproducible zone-appropriate marker expression matching the native reference panel, with hypertrophy markers (collagen type X, alkaline phosphatase) [150] benchmarked against native zonal references; (3) tissue construction, circumferential tensile modulus within a defined fraction of the native target (Table 3) and demonstrable collagen anisotropy, both as development targets rather than validated acceptance criteria; (4) functional maturation, maintenance of phenotype and mechanical properties under daily dynamic loading for a duration informed by precedent (for example at least three weeks [151]); (5) preclinical safety, no tumor formation in a tumorigenicity-sensitive in vivo model (for example NOG mice) over an observation period informed by precedent (for example 16 weeks [131]); and (6) large-animal validation, graft survival, root-fixation retention, resistance to extrusion and chondroprotection over a follow-up duration informed by precedent (for example ≥3 months in a large-animal model [152]). Failure at any gate redirects development before the complexity of a whole-construct therapy is increased.

## 10. Conclusions

When repair is not feasible, current options remain limited: partial meniscectomy relieves symptoms at the cost of load-bearing tissue, while allograft transplantation and acellular scaffolds carry their own constraints of supply, sizing or durability. None has been shown to restore native, functional meniscal tissue. iPSCs offer a route to change that: a renewable cell source already shown to engraft within an injured meniscus [31]. What remains unproven is whether that cartilage can become a meniscus: no current approach has generated zone-specific fibrochondrocyte identity, root-anchored architecture or durable chondroprotection in a reproducible way. Closing that gap requires treating iPSC differentiation biology, zonal meniscal identity and functional tissue reconstruction as one problem rather than three separate ones. The decisive test is therefore not whether iPSCs can form cartilage, but whether they can be directed into a tissue that behaves like a native meniscus under load. Until a construct clears that bar in clinically relevant models, the approach remains a compelling platform rather than an established therapy. The path from pluripotent versatility to anatomically faithful, functionally measurable, clinically deployable tissue remains the central translational challenge for an iPSC-derived meniscus.

## Figures and Tables

**Figure 1:**
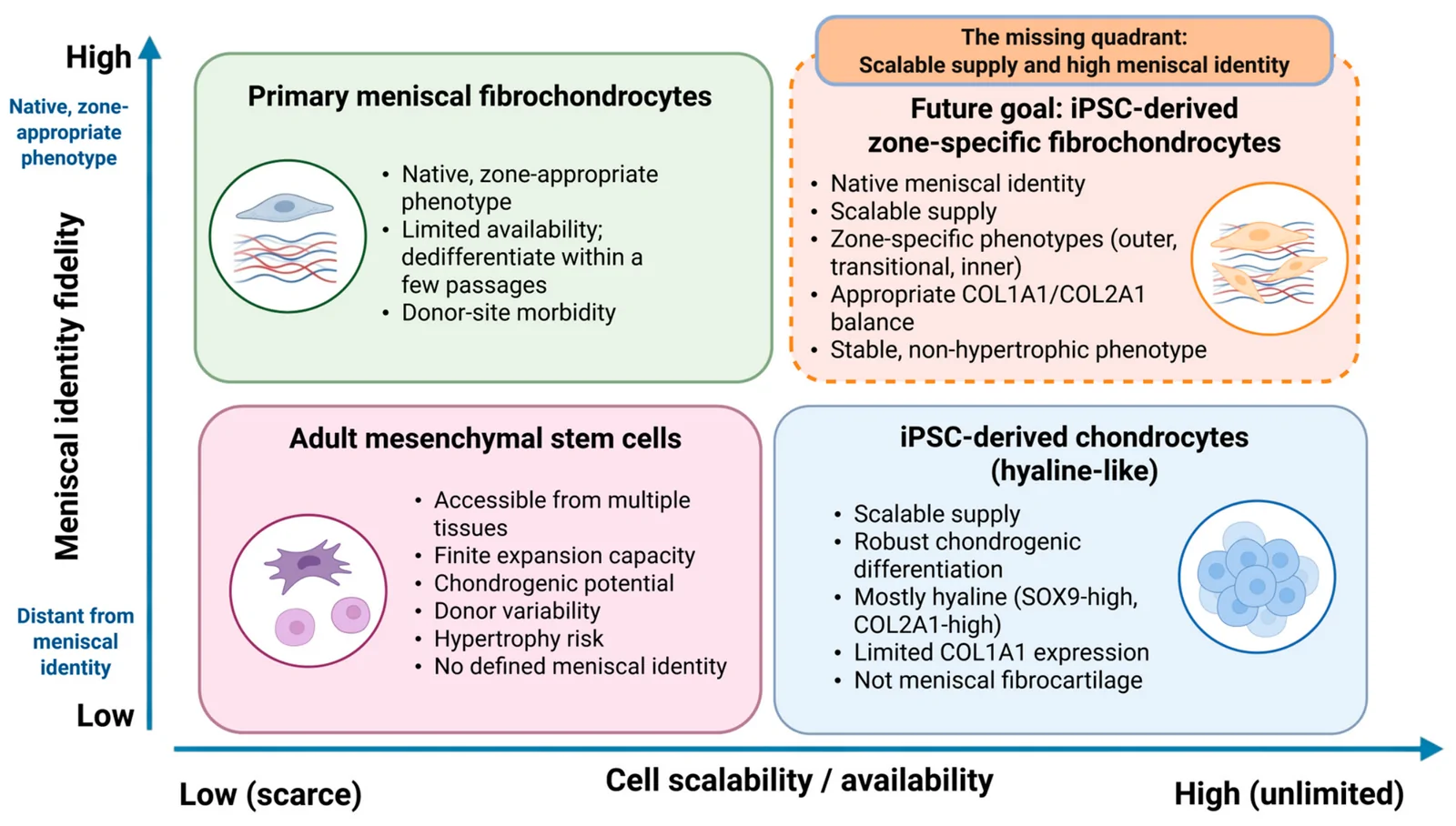
Candidate cell sources for meniscus regeneration, positioned by cell scalability and meniscal identity fidelity. Both axes are qualitative. Primary meniscal fibrochondrocytes have the correct phenotype but are scarce and dedifferentiate in culture. Adult MSCs are accessible but donor-variable, prone to hypertrophy and lack a defined meniscal identity. iPSC-derived chondrocytes are scalable but remain hyaline-biased. The upper right quadrant, iPSC-derived zone-specific fibrochondrocytes, is a target that has not yet been achieved. Created with BioRender.com (accessed on 21 October 2026).

**Figure 2:**
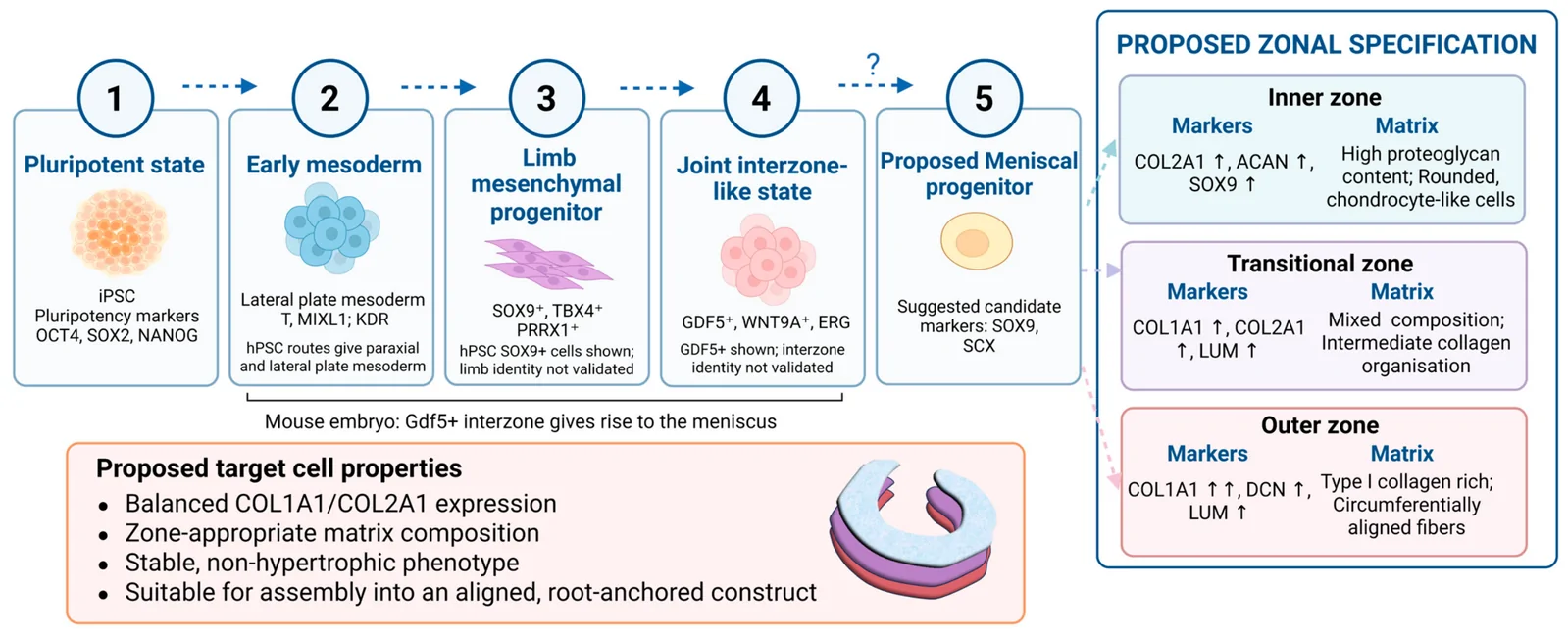
Proposed differentiation route from pluripotent cells toward a meniscal progenitor, with developmental zonal specification. Dashed arrows indicate steps shown in human pluripotent cells for cartilage or tendon differentiation, but not yet demonstrated toward the meniscus; the dotted arrow with “?” indicates an untested hypothesis. The GDF5-positive interzone origin of the meniscus is established by animal lineage tracing, not by evidence in human pluripotent cells; GDF5-positive cells derived from human pluripotent cells have so far shown only weak similarity to interzone cells, and the combined route to a meniscal progenitor, including titrated SCX and SOX9 activation, has not been tested. Proposed zonal marker panels and target cell properties are shown as development targets, not yet achieved. Arrows from the meniscal progenitor to the three zones indicate proposed zonal specification, not yet demonstrated; ↑ and ↑↑ denote higher and markedly higher relative expression. Created with BioRender.com (accessed on 20 October 2026).

**Figure 3:**
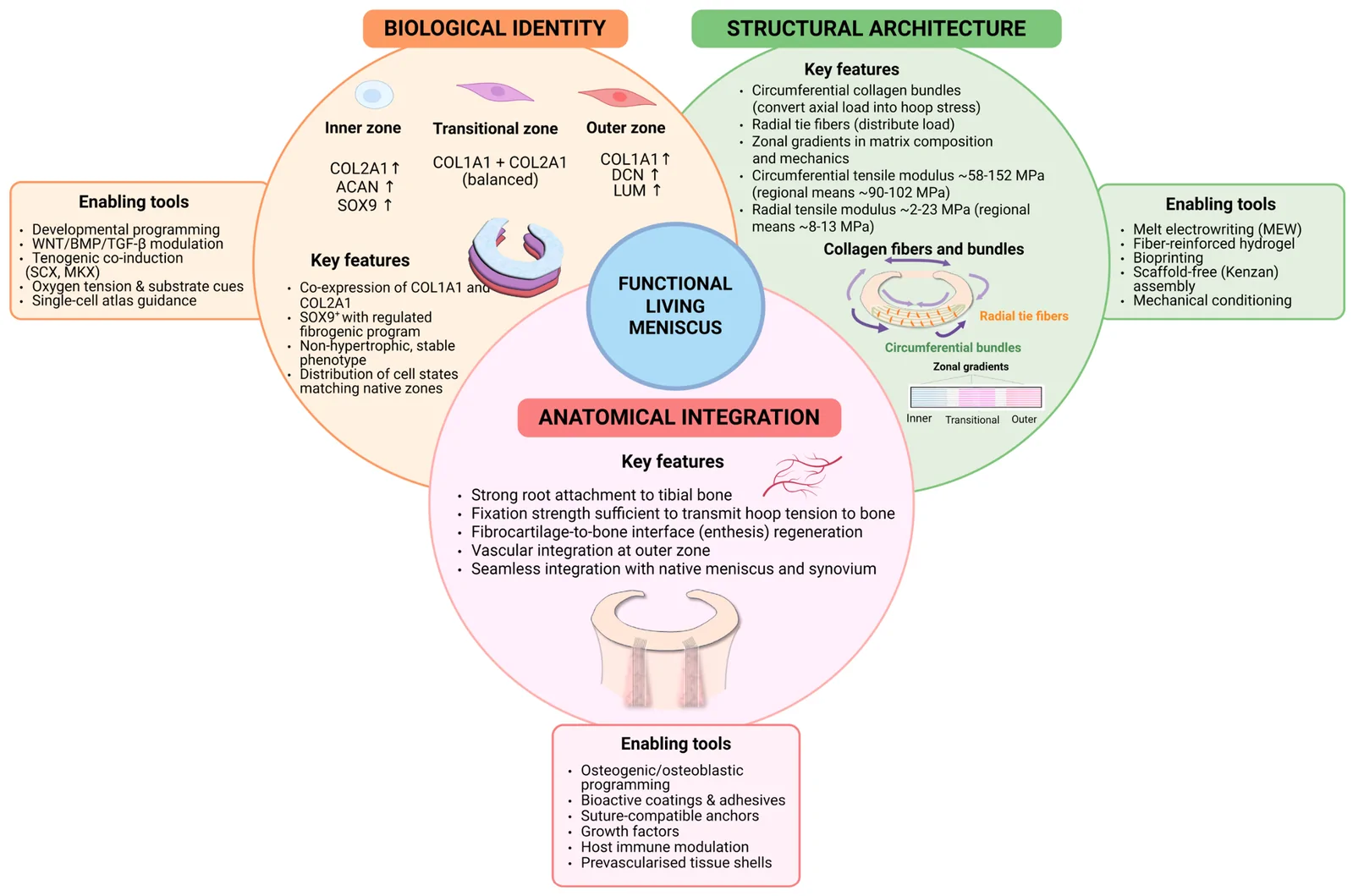
Three interdependent requirements for a functional living meniscus: biological identity (zone-specific fibrochondrocytes), structural architecture (aligned collagen and zonal gradients) and anatomical integration (root attachment, enthesis and vascularization). Enabling tools are shown outside each circle. Enabling tools are candidates, and most have not been tested with iPSC-derived meniscal cells. ↑ denotes higher relative expression; curved arrows in the collagen schematic indicate the direc-tion of circumferential collagen bundles. Created with BioRender.com (accessed on 20 October 2026).

**Figure 4:**
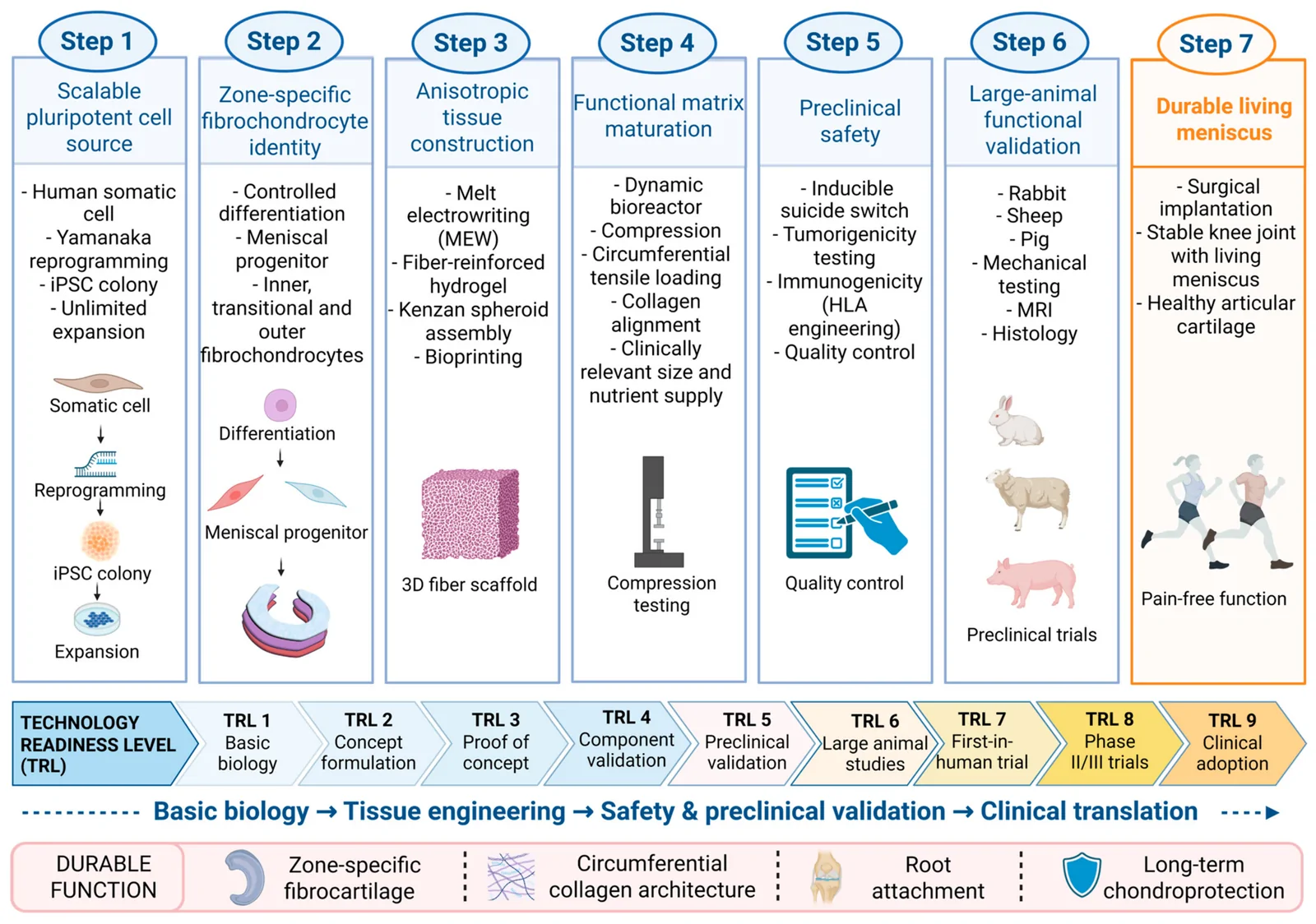
Proposed development pathway from pluripotent cells to a durable living meniscus. The seven milestones are a scalable pluripotent cell source, zone-specific fibrochondrocyte identity, anisotropic tissue construction, functional matrix maturation under dynamic loading, preclinical safety including inducible suicide-gene safeguards and tumorigenicity testing, large-animal functional validation with mechanical and histological endpoints, and a durable implant that restores joint function and protects articular cartilage. Only the first milestone is currently met. Created with BioRender.com (accessed on 20 October 2026).

**Table 2:** Five modalities for deploying iPSCs toward a meniscal implant, with maturity separated into three independent axes. Biological maturity refers to how close the deployed iPSC-derived cell is to a zone-specific meniscal fibrochondrocyte, rated low when no meniscal fate has been demonstrated, medium when a cartilaginous fate has been demonstrated in vivo, without confirmed long-term phenotypic stability, and high when zone-specific fibrochondrocytes have been produced and validated. Engineering maturity refers to the fabrication method irrespective of cell source, rated low for concept-stage methods, medium for methods validated with any cell type in vitro, and high for methods already used in patients or in large-animal implants. Translational maturity refers to the most advanced in vivo or clinical evidence for the relevant iPSC cell type. A modality can be engineering-mature and biologically immature, which is the current position of most device-based routes. Each maturity statement is tagged by evidence source: [D], direct iPSC-meniscus evidence; [C], iPSC-derived cartilage evidence; [I], indirect engineering or developmental evidence, including iPSC-derived evidence for non-meniscal tissues such as tendon; [H], no experimental data yet, so the statement is a hypothesis. Maturity refers to the level of experimental support, not to readiness for clinical use. iMSC, iPSC-derived MSC; MEW, melt electrowriting; ECM, extracellular matrix; PCL, polycaprolactone; GelMA, gelatin methacryloyl.

Modality	Basis and Evidence	Shape Control	Maturity on Three Independent Axes	Principal Risk
Direct (undifferentiated)	iPSCs or progenitors differentiate in situ; requires safety switches [55,56]	Low	Biological: low, no meniscal fate shown [H]. Engineering: low [H]. Translational: concept only [H]	Teratoma
Pre-differentiated	Implant chondrocytes, iMSCs or fibrochondrocytes [25,31,44,57]	Low to medium	Biological: medium; hyaline-derived particles engraft and give hyaline-rich or fibrous-rich repair in the rabbit meniscus, with no zone-specific fibrochondrocytes shown [D]. Engineering: medium [I]. Translational: small-animal meniscus, 4 weeks [31] [D]	Phenotype drift
Cell plus device	Printed PCL, decellularized meniscus ECM, or MEW fiber-reinforced hydrogel scaffolds to be seeded with iPSC-derived cells; these platforms have been shown acellularly or with non-iPSC cells [18,58,59,60]	High	Biological: low with iPSCs, no meniscal fate shown [H]. Engineering: medium to high [I]. Translational: acellular or non-iPSC scaffolds tested in animals or the clinic [I]; iPSC-seeded versions not yet shown in the meniscus [H]	Integration; residual DNA; poor infiltration
Bioprinting with ink	Cell-laden GelMA extrusion or inkjet; zonal gradients [58,61]	Medium to high	Biological: low with iPSCs, no meniscal fate shown [H]. Engineering: medium to high [I]. Translational: preclinical [I]	Weak; poorly aligned
Ink-free (Kenzan)	Spheroid needle-array fusion; iMSC cartilage in pigs [57,62,63]	Medium to high	Biological: medium with iMSCs [C]. Engineering: medium to high [I]. Translational: large-animal cartilage [61] [C]	Scale; maturation time

**Table 3:** Native human meniscus and engineered construct values with their test conditions. Moduli depend on loading mode, specimen orientation, strain rate, hydration, species and donor age, so rows are compared only where conditions are stated. No iPSC-derived meniscal construct has reported mechanical data; construct values are from cell-seeded or acellular scaffolds. Values are drawn from separate studies with different specimens and test protocols and are therefore a contextual benchmark rather than a matched head-to-head comparison. Differences in tissue region, loading direction, specimen preparation and test conditions may contribute to the reported variation. Conditions not specified in the original studies are not reported in the table.

Property	Native Meniscus Value Range	Typical Current Construct	Reference
Circumferential tensile modulus	57.97–151.80 MPa by region and layer; regional means 99.75 (anterior), 90.22 (central), 102.12 MPa (posterior). Uniaxial tension, 5 ± 1%/min, 95–100% humidity, ages 29–45	Aligned electrospun PCL, bovine meniscal cells, 10 wk: 19.7 MPa (0.1%/s); nonaligned: 5.0 MPa. Clamped collagen gels, bovine cells, 6 wk: ~1 MPa (0.75%/s). Acellular 3D-printed PCL: 18.2 ± 0.9 MPa (wet, 1 mm/min). Same loading mode; aligned PCL is about one-fifth of the regional means	[19,74,75,76]
Ultimate tensile strength (circumferential)	10.6 ± 8.4 MPa overall; 14.9 ± 9.9 MPa in donors <40 yr, 6.3 ± 3.3 MPa in donors >65 yr. Uniaxial tension to failure	Not reported for iPSC constructs	[77]
Failure strain (circumferential)	17.6 ± 4.5% grip-to-grip strain at ultimate stress (monotonic loading); 23.6 ± 6.8% in specimens that had undergone prior fatigue testing	Not reported for iPSC constructs	[77]
Radial tensile modulus	2.03–22.62 MPa; regional means 7.82, 11.49, 13.04 MPa. Uniaxial tension, 5%/min	Not reported for iPSC constructs	[75]
Compressive modulus	Unconfined, 12% strain: 76.1–83.4 kPa at equilibrium, 605–718 kPa at 32%/s. Confined aggregate modulus 0.11 ± 0.078 MPa (age 73.5 ± 6.4, PBS)	MEW PCL-reinforced GelMA: 214 ± 24 to 405 ± 68 kPa (unconfined, air, room temperature, 25%/min), versus ~7 kPa for gel alone. Acellular printed PCL: 380 ± 40 kPa (wet, 1 mm/min). Collagen constructs: equilibrium modulus ~50–70% of native (confined, 4 wk, bovine cells). Not matched: rate, bath and species differ	[71,76,78,79,80]
Collagen organization	Circumferential bundles with radial tie fibers	Predominantly random or unaligned	[3,19]
Root and enthesis fixation	Bony anchorage; avulsion or adjacent radial tear impairs hoop-stress transmission and raises contact pressure in cadaveric testing	Not addressed in current iPSC grafts; required for whole-meniscus replacement, not for focal-defect repair	[6,7]
Tensile fatigue resistance	Endurance limit ≈ 9–10% of ultimate tensile strength at 250 million cycles; S-N behavior spans ~500 cycles at 70% UTS to ~3 million cycles at 30% UTS (4 Hz, 37 °C saline bath). Donor-age effect disappears once stress is normalized to UTS	Not reported for iPSC constructs	[77]
Dynamic tensile modulus (viscoelastic behavior, outer third)	Lateral meniscus 134–144 MPa, medial meniscus 115–124 MPa across 0.01–1 Hz loading frequency; modulus increases with frequency; no correlation with donor age (15–44 yr)	Not reported for iPSC constructs	[81]
Vascular supply	Vascularized outer third and avascular inner region supplied by diffusion	Not demonstrated at clinically relevant implant scale	[3,66,67]

**Table 4:** Representative preclinical studies underpinning an iPSC-based meniscus, from foundational chondrogenesis to the first meniscal engraftment. The evidence source column states where the finding comes from: [D], direct iPSC-meniscus evidence; [A], adult-cell meniscus evidence; [C], iPSC-derived cartilage evidence; and [I], indirect engineering or developmental evidence, including iPSC-derived evidence for non-meniscal tissues such as tendon. The relevance to the meniscus column states what the finding supports, and where the link to the meniscus goes beyond what the study showed, the text says inferred or untested. iPSC, induced pluripotent stem cell; iMSC, iPSC-derived mesenchymal stem cell; scRNA-seq, single-cell RNA sequencing.

Focus	Model or System	Key Finding	Evidence Source	Relevance to Meniscus
Reprogramming [22,23]	Mouse and human fibroblasts	Defined-factor pluripotency	[I]	Provides a renewable, patient-matched cell source
Scaffold-free iPSC cartilage [25]	Human iPSC; mouse, rat and pig	Hyaline cartilage particles; survive and can integrate with native cartilage in joint-surface defects	[C]	Hyaline tissue only; a possible template for inner-zone tissue (inferred)
Primate engraftment [26]	Monkey (cynomolgus) iPSC; macaque chondral defect	Allogeneic iPSC cartilage organoids elicit no immune reaction on histology, integrate, repair for at least 4 months and acquire PRG4	[C]	Large-animal precedent for iPSC cartilage; not tested in the meniscus
Substrate and YAP control [45]	Human iPSC, in vitro	Culture substrate and YAP activity gate chondrogenesis; YAP knockdown or inhibition rescues it	[C]	Culture-condition lever on chondrogenesis; effect on zonal identity untested
scRNA-seq of iPSC chondrogenesis [47]	Human iPSC	WNT and MITF drive off-target fates and hypertrophy	[C]	Identifies signals to suppress for phenotype control
Protocol without BMPs [48]	Human iPSC, in vitro	Chondrocytes made without pro-hypertrophic BMPs show low hypertrophy and mineralization	[C]	Stable non-hypertrophic phenotype in vitro; in vivo stability untested
GDF5-positive chondroprogenitors [92]	Human pluripotent stem cells	Permanent-like, largely unmineralized cartilage with PRG4 after ectopic transplantation; teno/ligamentogenic potential suggested by transcriptomics	[C]	Candidate branch point for a meniscal program (inferred); the GDF5-positive cells showed only weak similarity to interzone cells
PRG4-positive cells [94]	Mouse iPSC	Superficial-zone and fibroblast-like cells induced at low efficiency (about 2.2%); survival 3 days in vivo	[I]	Shows non-hyaline joint fates can be induced; not shown to be stable
Tenogenic induction [98,99]	Human iPSC-derived MSCs (iMSCs)	SCX or MKX with cyclic stretch gives aligned collagen and ECM; the Mkx bio-tendon was tested in mouse Achilles rupture	[I]	Could supply the type I collagen arm of a fibrochondrocyte; combination untested
iMSC Kenzan cartilage [57]	Human iPSC-MSCs; immunodeficient minipig	Scaffold-free Kenzan patch implanted in osteochondral defects; human cells detected at the graft site	[C]	Ink-free construction route shown in cartilage; meniscal use untested
Primate meniscus regeneration [41]	Aged cynomolgus macaques; autologous synovial MSC aggregates after anterior-half medial meniscectomy	MSC group showed larger regenerated meniscus and better histological (Pauli) and cartilage (Mankin) scores than control in every animal; authors note no biomechanical testing was performed	[A]	Non-iPSC benchmark in the primate closest to humans; shows feasibility and chondroprotection, but not biomechanically validated
Rabbit meniscal defect repair [42]	Rabbit; 1.5 mm punch defect in avascular anterior horn; allogenic synovial MSC suspension	Greater and higher-quality regenerated tissue than control, with implanted cells differentiating into type-I/II collagen-producing cells resembling fibrochondrocytes; authors state biomechanical characteristics were not evaluated	[A]	Non-iPSC precedent for cell-mediated fibrochondrocyte-like differentiation in situ; same biomechanical gap as the iPSC study
Microminipig meniscal repair with biomechanical testing [50]	Microminipig; sutured longitudinal tear, avascular zone; allogenic synovial MSC suspension	Better healing by macroscopic, histological and T1rho MRI scores; tensile strength at failure was higher in the MSC group (10.5 N/mm^2^) than control (4.6 N/mm^2^), approaching intact meniscus (20.8 N/mm^2^)	[A]	The only adult-cell meniscus study in this set with biomechanical validation; sets a functional benchmark iPSC work has not yet reached
Microminipig degenerative-injury model [51]	Microminipig; novel 200-puncture degenerative injury model; allogenic synovial MSC suspension	Histological, immunohistochemical and T2-MRI improvement over control at 8 weeks; authors state no biomechanical analysis was performed	[A]	Non-iPSC evidence in a degeneration-mimicking model, closer to clinical presentation than a clean defect; same biomechanical gap as the iPSC study
First iPSC-meniscus study [31]	Human iPSC-derived cartilage particles; immunosuppressed rabbit meniscal-defect model; nine treated menisci and three autologous cartilage transplants; four-week follow-up	Untreated contralateral defects served as controls; autologous rabbit articular cartilage was used as a tissue-transplant comparator. Human cells were detected at the repair site; six of nine treated menisci showed hyaline-cartilage-rich repair and three showed predominantly fibrous repair. Outcomes were assessed histologically and by cell detection.	[D]	Direct evidence of short-term engraftment, but repair phenotype was variable; defects lay in the red-white zone; no quantitative scoring, biomechanical testing or long-term durability assessment

**Table 5:** Clinical-translation landmarks and product classes relevant to an iPSC meniscus. MAT, meniscal allograft transplantation; CMI, collagen meniscus implant; IMRC, immunity-and-matrix-regulatory cell; hESC, human embryonic stem cell.

Product or Study	Status	Setting	Relevance to iPSC Meniscus
Allogeneic iPSC-derived cartilage (jRCTa050190104) [29]	First-in-human; 4 patients (1 discontinued, unrelated AE); no tumorigenesis in 3 followed to 12 months	Articular cartilage defect	Nearest precedent for an iPSC-derived joint tissue
hESC-derived IMRCs, intra-articular injection [30]	Completed phase I, open-label, non-randomized, dose-escalation trial; 18 patients; three dose groups; 48-week follow-up	Meniscal injury; intra-articular cell injection	Early clinical safety data and exploratory MRI/knee-function outcomes; no placebo or untreated clinical control. A paracrine/immunomodulatory cell-therapy approach, not a structural meniscal graft.
iPSC-derived meniscal graft [31]	None to date; small-animal proof of concept	Meniscal defect	The unmet clinical gap addressed by this review
CMI and Actifit acellular scaffolds [16,17]	Marketed and in trials	Partial meniscal loss with intact rim and horn attachments [16]	Acellular benchmarks with variable durability
Meniscal allograft (MAT) [11]	Established	Total or near-total meniscus loss [2]	Geometry restoration; supply-limited

**Table 6:** Focal-defect repair, partial replacement and whole-meniscus replacement: requirements for an iPSC-derived product.

Requirement	Focal-Defect Repair	Partial Replacement	Whole-Meniscus Replacement
Setting	Contained tear or defect; roots and rim intact [2]	Resected segment; intact peripheral rim and both horn attachments [2,16,64]	Total or near-total meniscectomy [2]
Root fixation	Host roots remain	Host horn attachments must be intact; scaffolds sutured to the rim; Actifit studies excluded root tears [16]	Required at both roots; horn fixation with soft tissue, bone plugs or bone bridge; no technique shown superior [10]. Bone fixation restores contact mechanics better in cadaveric tests, but clinical outcomes and complications do not differ significantly [12,129]
Integration	Defect margin	Peripheral rim; scaffolds rely on the vascularized rim [64]; no solid rim connection in a sheep model [65]	Both roots and periphery; viability at centimeter scale
Vascularization/nutrient supply	Millimeter-scale graft; relies on native perilesional vascularity and diffusion from synovial fluid [3]	Centimeter-scale segment; depends on secure integration with an intact, vascularized peripheral rim [16,65]	Full centimeter-scale construct; must achieve peripheral vascular integration of its own or tolerate prolonged avascular survival in its inner region [66,67]
Construct size	Millimeter-scale, contained defect [3]	Centimeter-scale segment [16,65]	Full centimeter-scale, anatomically graded construct [66,67]
Mechanical demands	Local stability and integration with the defect margin [7]	Integration with the host rim and load transfer across the replaced segment [65]	Circumferential tensile load across the entire structure, tethered through the roots [6]
Delivery	Particle placement or injection [30,31]	Arthroscopic insertion is possible; suture fixation is an extensive procedure [64]; implants are hard to handle and suture [2]	Size-matched graft; mini-open or arthroscopic [10]
Precedent	Suture or anchor repair [2]	CMI and Actifit: failure 6.5% and 18% [16]	Allograft: survival free of failure 85% at 10 years and 72% at 15 years [11]
iPSC evidence	Small-animal engraftment only [31]	None	None

## Data Availability

No new data were created or analyzed in this study. Data sharing is not applicable to this article.

## Abbreviations

The following abbreviations are used in this manuscript:

ACAN	Aggrecan
ACL	Anterior cruciate ligament
ACTA2	Actin alpha 2, smooth muscle
AKS	American Knee Society (score)
ALP	Alkaline phosphatase
ALPL	Alkaline phosphatase, liver/bone/kidney type
ATMP	Advanced therapy medicinal product
bFGF	Basic fibroblast growth factor
BMP	Bone morphogenetic protein
CAT	Committee for Advanced Therapies
CCL20	C-C motif chemokine ligand 20
CD49b/CD49c	Integrin alpha-2/alpha-3 subunits
CD166	Cluster of differentiation 166
CHAD	Chondroadherin
CILP	Cartilage intermediate layer protein
CMI	Collagen Meniscus Implant
COL1A1	Collagen type I alpha 1 chain
COL2A1	Collagen type II alpha 1 chain
COL3A1	Collagen type III alpha 1 chain
COL10A1	Collagen type X alpha 1 chain
COMP	Cartilage oligomeric matrix protein
ddPCR	Droplet digital PCR
DCN	Decorin
ECM	Extracellular matrix
EMA	European Medicines Agency
EREG	Epiregulin
ERG	ETS-related gene
ESC	Embryonic stem cell
ESRG	Embryonic stem cell-related gene
FDA	U.S. Food and Drug Administration
FIDELITY	Finnish Degenerative Meniscal Lesion Study
FMOD	Fibromodulin
FOXD3	Forkhead box D3
GDF5	Growth differentiation factor 5
GelMA	Gelatin methacryloyl
GMP	Good manufacturing practice
hESC	Human embryonic stem cell
HIF-1α	Hypoxia-inducible factor 1 alpha
HLA	Human leukocyte antigen
IBSP	Integrin-binding sialoprotein
ICRS	International Cartilage Repair Society
IFN-γ	Interferon gamma
IHH	Indian hedgehog
IKDC	International Knee Documentation Committee
IL-1β	Interleukin-1 beta
IMRC	Immunity-and-matrix-regulatory cell
iMSC	Induced pluripotent stem cell-derived mesenchymal stem cell
iPSC	Induced pluripotent stem cell
ISAKOS	International Society of Arthroscopy, Knee Surgery and Orthopedic Sports Medicine
jRCT	Japan Registry of Clinical Trials
KDR	Kinase insert domain receptor
KOOS	Knee injury and Osteoarthritis Outcome Score
LIN28A	Lin-28 homolog A
LINC00678	Long intergenic non-protein coding RNA 678
LUM	Lumican
MAT	Meniscal allograft transplantation
METEOR	Meniscal Tear in Osteoarthritis Research
MHC	Major histocompatibility complex
MITF	Melanocyte inducing transcription factor
MIXL1	Mix paired-like homeobox
MKX	Mohawk homeobox
MMP1/MMP2/MMP3/MMP13	Matrix metalloproteinase 1/2/3/13
MOCART	Magnetic Resonance Observation of CArtilage Repair Tissue
MRI	Magnetic resonance imaging
MSC	Mesenchymal stem cell
NANOG	Nanog homeobox
NES	Nestin
NOG	Immunodeficient mouse strain
OCT4	Octamer-binding transcription factor 4
OGN	Osteoglycin
OTX2	Orthodenticle homeobox 2
PAX3	Paired box 3
PAX6	Paired box 6
PCL	Polycaprolactone
PECAM1	Platelet endothelial cell adhesion molecule 1
PLVAP	Plasmalemma vesicle-associated protein
PRELP	Prolargin (proline/arginine-rich end leucine-rich repeat protein)
PRG4	Proteoglycan 4
PRRX1	Paired related homeobox 1
PTH1R	Parathyroid hormone 1 receptor
RMAT	Regenerative Medicine Advanced Therapy
RUNX2	Runt-related transcription factor 2
scRNA-seq	Single-cell RNA sequencing
SCX	Scleraxis
SOX2	SRY-box transcription factor 2
SOX9	SRY-box transcription factor 9
TAGLN	Transgelin
TBX4	T-box transcription factor 4
TGF-β	Transforming growth factor beta
THBS4	Thrombospondin 4
TNF-α	Tumor necrosis factor alpha
TNFAIP6	TNF alpha-induced protein 6
TRL	Technology readiness level
VAS	Visual analog scale
VWF	Von Willebrand factor
WNT	Wingless-related integration site
WOMAC	Western Ontario and McMaster Universities Osteoarthritis Index
YAP	Yes-associated protein
ZIP8	Zinc transporter 8 (SLC39A8)
